# Diverse lifestyles and adaptive evolution of uncultured UBA5794 actinobacteria, a sister order of “*Candidatus* actinomarinales”

**DOI:** 10.1186/s40793-025-00701-w

**Published:** 2025-04-19

**Authors:** Jing Huang, Xiaowei Zheng, Tao Yu, Mohsin Ali, Jutta Wiese, Songnian Hu, Li Huang, Ying Huang

**Affiliations:** 1 State Key Laboratory of Microbial Diversity and Innovative Utilization, Institute of Microbiology, Chinese Academy of Sciences, Beijing, China; 2 College of Life Sciences, University of Chinese Academy of Sciences, Beijing, China; 3 RU Marine Ecology, RD3 Marine Symbioses, GEOMAR Helmholtz Centre for Ocean Research Kiel, Kiel, Germany; 4 Southern Marine Science and Engineering Guangdong Laboratory (Guangzhou), Guangzhou, 511458 China

**Keywords:** UBA5794, Metagenome, Metabolism, Habitat adaptation, Evolution, Actinobacteria, *Acidimicrobiia*

## Abstract

Uncultured UBA5794 actinobacteria are frequently found in marine and inland water environments by using metagenomic approaches. However, knowledge about these actinobacteria is limited, hindering their isolation and cultivation, and they are always confused with “*Candidatus* Actinomarinales” based on 16S rRNA gene classification. Here, to conduct genomic characterization of them, we obtained three high-quality UBA5794 metagenome-assembled genomes (MAGs) from a hydrothermal sediment on the Carlsberg Ridge (CR) and retrieved 131 high-quality UBA5794 genomes from public datasets. Phylogenomic analysis confirms UBA5794 as an independent order within the class *Acidimicrobiia*. Genome-based metabolic predictions reveal that flexible metabolism and diversified energy acquisition, as well as heavy metal(loid) detoxification capacity, are crucial for the ability of UBA5794 to thrive in diverse environments. Moreover, there is separation between sponge-associated and free-living UBA5794 groups in phylogeny and functional potential, which can be attributed to the symbiotic nature of the sponge-associated group and the extensive horizontal gene transfer (HGT) events observed in these bacteria. Ancestral state reconstruction suggests that the UBA5794 clade may have originated from a free-living environment and then some members gradually migrated to the sponge host. Overall, our study sheds light on the ecological adaptation and evolutionary history of the ubiquitous but poorly understood UBA5794 actinobacteria.

**Supplementary Information:**

The online version contains supplementary material available at 10.1186/s40793-025-00701-w.

## Background

Uncultured taxa of the phylum *Actinobacteria* have been extensively detected in aquatic ecosystems with the advent of 16S rRNA gene and metagenomic sequencing. In one of the earliest studies, nine fragmentary sequences representing a deep-branching clade were discovered from the 16S rRNA gene clone libraries of the Pacific and Atlantic Oceans [1]. This deep-branching clade was then found to be highly similar to marine 16S rRNA gene clades of OM1 and OM231 from the Atlantic Ocean and SAR432 from the Sargasso Sea [2]. A follow-up study based on full-length 16S rRNA gene sequences prompted the suggestion that the above clades, together with a marine bacterioplankton (OCS155), should represent a subclass or order within *Actinobacteria*, which was referred to as the marine *Actinobacteria* clade (MAC) [3].

Subsequently, a group of marine *Actinobacteria* with very low GC content was uncovered from the Mediterranean deep chlorophyll maximum [4]. Phylogenetic analysis of the complete 16S and 23S rRNA gene sequences retrieved from the assembled metagenomic contigs indicated that this group was highly related to the previously described MAC, leading to the proposal of a new order, “*Candidatus* Actinomarinales” within the class *Acidimicrobiia* [4]. Recently, members within this order were reclassified and divided into five genera and 18 genomic species by comparing and analyzing their single-amplified genomes (SAGs) and metagenome-assembled genomes (MAGs) [5]. These genomospecies showed a preference for the photic zone of the ocean and had extremely small (estimated to be ~ 1.1 Mb) genomes with a low GC content (estimated to be ~ 32.5%) [5]. Their highly streamlined genomes were featured by a small number of sigma factors and strikingly short intergenic spacers, as well as by the absence of identifiable mobile genetic elements, toxin-antitoxin, or CRISPR systems [5]. In addition, two types of rhodopsins, Type 1 (MACRhodopsin) and Type 3 (heliorhodopsin), were detected in their genomes, suggesting a photoheterotrophic lifestyle [5].

During our previous investigation of the diversity of *Actinobacteria* in deep-sea sediments from the Southwest Indian Ridge (SWIR) and Carlsberg Ridge (CR), we found that up to 55% of the total actinobacterial sequences belonged to the OM1 clade of MAC, based on taxonomic assignments from the SILVA 128 database [6]. At the beginning of this study, we performed full-length 16 S rRNA gene amplicon sequencing on the sample N04 that had the highest relative abundance of OM1 clade and found that the clade was assigned to “*Ca*. Actinomarinales” using updated SILVA 138.1 database. However, phylogenetic analysis of the full-length 16 S rRNA sequences revealed an obvious separation between our clade and previously recovered “*Ca*. Actinomarinales”. Further phylogenomic analysis showed that our clade was affiliated to UBA5794 order based on Genome Taxonomy Database (GTDB), probably forming a sister order to “*Ca*. Actinomarinales” within the class *Acidimicrobiia*. This observation raised our concern about the inaccurate designation of the environmental amplicon sequences as “*Ca*. Actinomarinales” or OM1 clade and the resulting misunderstanding of their ecological functions.

Up till now, compared to the systematic studies on “*Ca*. Actinomarinales”, there still exist knowledge gaps in terms of reliable taxonomic placement and functional traits of UBA5794. UBA5794 was formally named and included in the UBA (Uncultivated Bacteria and Archaea) dataset consisting of nearly 8,000 MAGs recovered from publicly available metagenomes [7]. Most UBA5794 genomes were frequently detected in the metagenomes of aquatic environments, including deep-sea hydrothermal sediments [8, 9], Challenger Deep sediments [10], beach sand [11], sponges [12] and fresh/groundwater [13]. Besides, a scatter of UBA5794 groups were also discovered in hyperarid desert [14], dinosaur fossil [15], the isolated Movile Cave [16], permafrost [17] and marine ANAMMOX bioreactor [18]. In contrast with planktonic “*Ca*. Actinomarinales”, UBA5794 taxa appear to be associated with sessile forms, occupying a wider range of ecological niches in diverse oligotrophic, harsh and symbiont environments. This aroused our interests in their survival strategies and evolutionary processes. In this study, through metagenomic and comparative genomic analyses, we provide deeper insights into the accurate phylogenetic position, comprehensive metabolic characteristics, habitat adaptability, and the evolution process of the poorly understood UBA5794 actinobacteria.

## Materials and methods

### Sample collection, enrichment, and DNA extraction

The sediment sample N04 (2986 m, 60.53° E, 6.36° N) was collected from the Wocan hydrothermal field on the CR using TV-guided grabs during the DY-28I cruise of the Haiyang 20 research vessel [19, 20]. The sample was transferred into sterile 200-ml plastic boxes and frozen at -80 °C until further processing in the laboratory.

Before extraction of genomic DNA, the sample was enriched using oligotrophic SN liquid culture medium, which was recently applied to isolate from sea sediments a distinct member, *Actinomarinicola tropica* gen. nov., within the *Acidimicrobiia* class [21]. Briefly, 2.5 g of sediment was inoculated into 45 mL SN medium in a 50-ml centrifuge tube, and then incubated in the dark at 10 °C without shaking for 40 days. After centrifuging the enriched sample at 7000 rpm for 10 min, the supernatant was discarded and 0.5 g pellet was taken for the total genomic DNA extraction using a DNeasy PowerSoil Kit (QIAGEN, GmbH, Hilden, Germany) according to the manufacturer’s instructions. The concentration of total genomic DNA was measured by Qubit 2.0 Fluorometer (Invitrogen, Carlsbad, CA, USA) and the quality was additionally checked by gel electrophoresis.

### 16S rRNA gene amplicon sequencing and analysis

The full-length 16S rRNA genes of enriched N04 samples was amplified using the universal bacterial primer set 27F (5’-AGAGTTTGATCCTGGCTCAG-3’) and 1492R (5’-GGTTACCTTGTTACGACTT-3’) and sequenced at Novogene Co. Beijing using PacBio single molecule sequencing technique (SMRT). The raw reads were first demultiplexed according to their unique barcode by the lima software and then corrected using SMRT Link v7.0. The resulting clean sequences were assigned to operational taxonomic units (OTUs) at a similarity cutoff of 97% by UPARSE [22] and representative sequences of the OTUs were classified based on SILVA 138.1 [23] using the Mothur software [24].

### Metagenomic sequencing and assembly

Paired-end sequencing (2 × 250 bp) of genomic DNA extracted from enriched sample N04 was performed on Illumina Hiseq-2500 platform at the Institute of Microbiology, Chinese Academy of Sciences (CAS), Beijing. Metagenome raw reads were trimmed and low-quality sequences (Phred quality score < 30) were removed using Trim-galore (v0.4.0). Then a total of 10 Gbp clean reads were merged and assembled by metaSPAdes (v3.9.0) [25] with the following kmer sets: 21, 29, 39, 59, 79, 99, 127. For the subsequent genome binning procedure, the scaffolds shorter than 500 bp were removed. Genome binning was performed by Concoct (v1.0.0), MetaBAT2 (v2.12.1) and MaxBin2 (v2.2.5) methods in MetaWRAP [26] pipeline with default parameters. Taxonomy of the resulting MAGs was assigned based on the GTDB using GTDB-Tk (v2.0.0) [27], followed by the genome size estimation and coding density calculation, as well as by genome completeness, contamination, and strain heterogeneity evaluation through CheckM (v1.0.9) [28].

### Refinement of *Acidimicrobiia* MAGs

Considering the possibility of high heterogeneity of *Acidimicrobiia* MAGs, we attempted to sample partial sequencing data (10% and 50%) of the enriched N04 sample to recover *Acidimicrobiia* MAGs with high quality. Resampled sequences were assembled using both Megahit (v1.2.9) [29] and SPAdes (v3.13.1) [30] software, and two sets of kmers (27, 33, 55, 65, and 21, 33, 55, 77, 99127) were configured when running SPAdes, followed by genome binning as described above. As expected, resampling of 1 Gbp clean reads was able to improve the genomic quality of *Acidimicrobiia* MAGs. The redundant bins were removed at an average nucleotide identity (ANI) threshold of 0.99.

### Phylogenetic analysis

For an overall understanding of the phylogenetic relationship between UBA5794 and “*Ca*. Actinomarinales”, 2349 reference 16S rRNA gene sequences of “*Ca*. Actinomarinales” with high quality (pintail quality > 75%, sequence length > 1,000 nucleotides, sequence quality > 75%) were collected from the SILVA 138.1 database. Alignment of the obtained reference sequences combined with sequences from this study was performed by MAFFT (v7.505) (AUTO model) [31]. Gblocks (v0.91b) was used to trim the alignments by removing poorly aligned positions and divergent regions with the following parameters: Gblocks -b4 = 5 -b5 = h [32]. The 16S rRNA gene phylogenetic tree was inferred using the maximum-likelihood (ML) approach in MEGA11 [33] or FastTree (v2.1.3) (GTR model) [34].

All the available genomes annotated as UBA5794 as well as representative genomes from major genera of the order “*Ca*. Actinomarinales” [5], other orders of the class *Acidimicrobiia*, and related classes of the phylum *Actinobacteria* were downloaded from the GTDB (Release 214) database. These genomes were analyzed together with the UBA5794 MAGs recovered in this study and our previous study (TVG06 and TVG10 bins) [35]. A total of 134 high-quality UBA5794 genomes (completeness > 70%, contamination < 10%) were collected. After removing redundant genomes based on an ANI threshold of 0.99, 98 genomes were selected for phylogenetic inference and subsequent functional and evolutionary analyses. Concatenated alignment of 120 bacterial marker proteins produced by GTDB-Tk was used to build a phylogenomic tree using IQ-tree (v2.1.2) (parameters: iqtree -m MFP -bb 1000 -bnni -nt AUTO) [36].

To accurately clarify the phylogenetic structure within UBA5794, a sub-phylogenomic tree was constructed based on protein-coding sequences (CDSs) files of high-quality genomes, outgrouped with representatives of “*Ca*. Actinomarinales”. All CDSs were clustered using OrthoFinder [37] algorithms to generate orthogroups (OGs) and using DIAMOND for sequence alignment with E-values < 1e − 5. The longest sequence of each OG was chosen and annotated by DIAMOND using the non-redundant protein sequence (NR) database with E-values < 1e − 5 [38]. Then, IQ-tree (v2.1.2) [36] was used as above to generate phylogenomic tree based on 291 OGs, with a minimum of 80% de-redundant genomes from UBA5794 and “*Ca*. Actinomarinales” having single-copy CDSs in each OG. All trees were visualized using iTOL (https://itol.embl.de/).

### Genome annotation and metabolic reconstruction

CDSs were predicted using Prodigal software in the Prokka [39] pipelines with default parameters, and then annotated against Kyoto Encyclopaedia of Genes and Genomes (KEGG) database using Kofamscan to assign KEGG Orthologs (KOs) to CDS. Meanwhile, HMMscan search was performed against the PfamA database [40] with E-values < 1e − 5. Carbohydrate-active enzymes (CAZy) and peptidases annotations were conducted by METABOLIC-C.pl program in the METABOLIC (v2.0) [41] annotation pipeline. Hydrogenases were further checked and classified based on the hydrogenase classifier HydDB web tool (https://services.birc.au.dk/hyddb/). To explore the specific function of CAZy family shown in UBA5794, all corresponding protein sequences were retrieved and then searched against the NR database by local blast with E-values < 1e − 5.

### Identification of habitat-associated genes/domains

Cluster results of KOs, PFAMs, and OGs were selected to identify habitat-associated genes/domains. For the subsequent statistical analysis, clusters with single counts were filtered. Bray-Curtis distance matrices was used to measure the dissimilarities of gene content composition, and the principal coordinate analysis (PCoA) was used to visualize the functional differences, both carried out with the vegan package in R v4.0.2 (https://www.rproject.org/). Scoary [42] was used to perform habitat-associated gene enrichment analysis, using the gene presence/absence datasets generated from the above three clustering methods. A gene cluster was considered significant by Scoary only if it had a p-value less than 0.05 for Fisher’s exact test. P-values were corrected with Benjamini-Hochberg FDR for multiple comparisons, using q < 0.05 as the significance threshold. And odds ratio above 1 and less than 1 were consider as present in habitat-positive (free-living) and habitat-negative (sponge) environment, respectively.

### Horizontal gene transfer (HGT) identification

HGTector2 was employed to identify genome-wide HGT events [43] among UBA5794. The first step in HGTector2 was to perform sequence similarity searches for input protein sequences against the NR database to find homologous sequences (evalue 1e-5; minsize 50; identity 30). Then, putatively HGT-derived genes were analyzed using the output results from the searching step. Since all the UBA5794 groups are uncultured, taxonomic classification of the proteins in the database is always assigned only to the rank of class *Acidimicrobiia*. Therefore, the taxonomic name of the “input genome” and “self” group was specified as *Acidimicrobiia*. *Actinobacteria* was designated as the “close” group and all others constituted the “distal” group, both of which were potential HGT donors to UBA5794.

### Reconstruction of ancestral lifestyles

Ancestral state reconstruction of the habitat and tracing of habitat evolution across the UBA5794 clade were performed using Mesquite v3.31 (available at http://mesquiteproject.org), based on the sub-phylogenomic tree of UBA5794 and observed habitat distribution (e.g., sponge-associated and free-living) within the clade. The probability of distinct habitat states was calculated using the ML reconstruction method with Markov k-state one-parameter (Mk1, for multistate data) and asymmetrical two-parameter Markov k-state (Asymm.2, for binary data) models.

## Results and discussion

### Phylogenetic placement of UBA5794

**Fig. 1:**
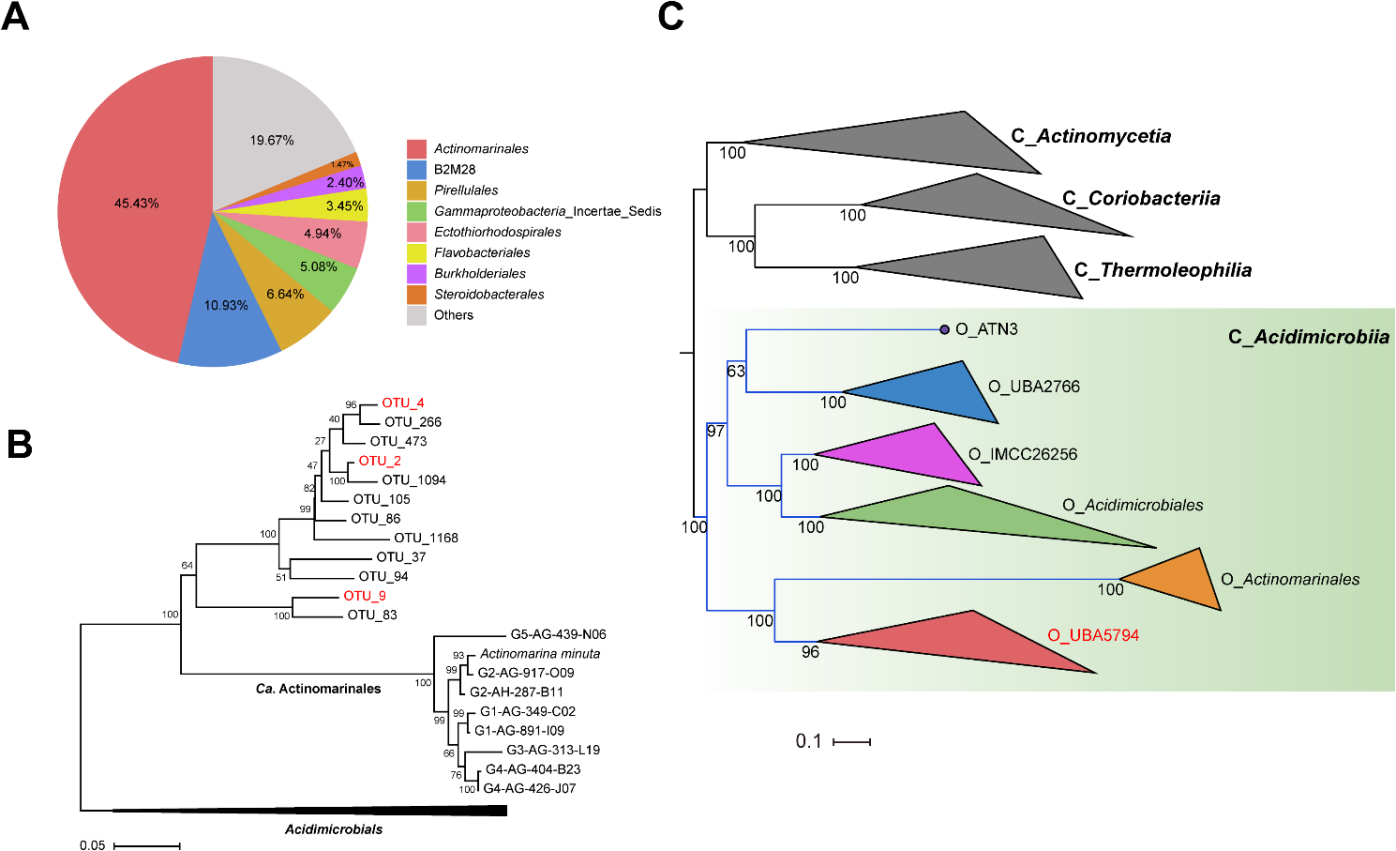
Phylogenetic analysis of uncultured UBA5794 actinobacteria. (**A**) Prokaryotic community composition at the order level (relative abundance > 1%) of the enriched sample of deep-sea hydrothermal sediment N04, based on 16S full-length rRNA gene amplification. (**B**) The maximum-likelihood (ML) phylogenetic tree based on 16S rRNA genes of the 12 main OTUs obtained in this study and those from representative “*Ca*. Actinomarinales” MAGs. The tree was constructed with MEGA11. Three dominant OTUs (OTU2, 4, and 9) are shown in red font. (**C**) Genome-wide ML phylogenetic analysis of the class *Acidimicrobiia* based on the 120 bacterial concatenated marker proteins identified by GTDB-Tk. All the orders of *Acidimicrobiia* by the GTDB taxonomy are included in this phylogenomic tree, with outgroups of nearby actinobacterial classes (*Actinomycetia*, *Coriabacteriia*, and *Thermoleophilia*). The tree was constructed using IQ-tree (v2.1.2) with parameters: iqtree -m MFP -bb 1000 -bnni -nt AUTO, and the branches are collapsed into triangles at the order or class level

According to the full-length 16S rRNA gene amplicon sequence data classified based on SILVA 138.1, the “*Ca*. Actinomarinales” order of the *Acidimicrobiia* class was particularly abundant in the enriched N04 sample, accounting for 45.43% of the prokaryotic community (Fig. 1A). The sequences annotated as “*Ca*. Actinomarinales” were binned into 12 non-singleton OTUs at the 97% sequence similarity cutoff, with three OTUs (OTU_2, 4, and 9) having relative abundance greater than 1% (Table S1).

However, the phylogenetic tree based on 16S rRNA gene manifested a separation between the branch composed of the 12 OTUs and that of the previously described order “*Ca.* Actinomarinales” (Fig. 1B). The two branches shared sequence identity ranging from 82.64 to 86.44%, which was below the 89.20% median sequence identity at the order level [44], implying that the OTUs might be distinct from “*Ca.* Actinomarinales”. To verify this inference, we collected all high-quality 16S rRNA gene sequences of “*Ca.* Actinomarinales” from the SILVA 138.1 database to perform a phylogenetic classification. The phylogeny showed that the clade annotated as “Actinomarinales; Actinomarinaceae” differed obviously from the other clade annotated as “Actinomarinales; uncultured” and containing the 12 OTUs (Fig. S1). The sequence identity between the two clades ranged from 79.68 to 88.39%, with all the pairwise comparisons being less than 89.20%. Briefly, phylogenetic analysis of “*Ca.* Actinomarinales” 16S rRNA gene sequences implies the possibility that “Actinomarinales; uncultured” forms a separate order from the “Actinomarinales; Actinomarinaceae” clade.

For a sophisticated phylogenomic designation, we performed metagenome sequencing of enriched N04 to recover MAGs containing 16S rRNA gene sequences in coincidence with OTUs_2, 4, and 9. Metagenome assembly and binning of 10 Gbp clean sequencing data (Table S2) resulted in 38 MAGs (completeness > 50% and contamination < 10%), including five MAGs assigned to the class *Acidimicrobiia* (three UBA5794 and two *Acidimicrobiales* MAGs based on the GTDB classification) (Table S3). Two 16S rRNA sequences were obtained from the UBA5794 MAGs (metabat2_bin.29 and metabat2_bin.28), which showed 100% identity and clustered in highly supported terminal branches with OTU_2 and OTU_9, respectively (Fig. S2). This result indicates that the two OTUs were amplified from members of UBA5794 in the sample.

After refinement and reassembly of UBA5794 bins, three MAGs with high quality were recovered (Table S4). We also collected available high-quality UBA5794 genomes from the GTDB Release 214 (122 genomes) and our previous study (9 genomes) [35] (Table S4). After removing redundant MAGs at a 99% ANI threshold, the resulting 98 MAGs, as well as genomes corresponding to other orders of the class *Acidimicrobiia*, were used to construct a phylogenomic tree. The phylogenomic analysis corroborated the stand-alone placement of UBA5794 in parallel with “*Ca*. Actinomarinales” (Fig. 1C; Fig. S3). Coupling with the phylogenetic analysis of 16S rRNA gene sequences described above, we propose that the “Actinomarinales; uncultured” populations should be classified into an individual order (UBA5794) sister to “*Ca*. Actinomarinales”. Recently, the decorated taxonomy frame in Greengenes2, which unifies genomic and 16S rRNA databases [45], has assigned the “UBA5794” order for 16S rRNA gene sequences of an uncultured group, further supporting our proposal.

### Genomic characteristics and habitat distribution

The genome sizes of the order UBA5794 were 1.39 ~ 5.38 Mb with a median of 2.79 Mb, and the genomic GC content ranged from 59.10 to 72.50% with a median of 64.21% (Table S4). Unlike “*Ca*. Actinomarinales”, which have a much smaller genome size (ca. 1.1 Mb) and a much lower GC content (ca. 32.5%) [5], the high GC content of UBA5794 genomes is consistent with the overall genomic characteristics of *Actinobacteria*. Among all the 134 UBA5794 genomes, 52 were derived from marine sediments (including 28 genomes from hydrothermal sediments), 27 from sponges, 18 from beach sand, 11 from brackish sediments (river-sea transition zones), 10 from inland water systems, 4 from desert, and 12 from other environments (such as permafrost, dinosaur fossils, and the isolated Movile Cave) (Table S4). In comparison, most “*Ca*. Actinomarinales” taxa have a particular preference for the upper layers of the epipelagic ocean [5]. Collectively, UBA5794 actinobacteria exhibit remarkable diversity in genomic features, ecological distribution, and lifestyle, spanning marine to terrestrial habitats and free-living to symbiotic modes of existence. This diversity suggests the evolution of complex metabolic pathways to enhance their adaptability to varied environments.

### Central carbon metabolism and carbohydrate utilization

**Fig. 2:**
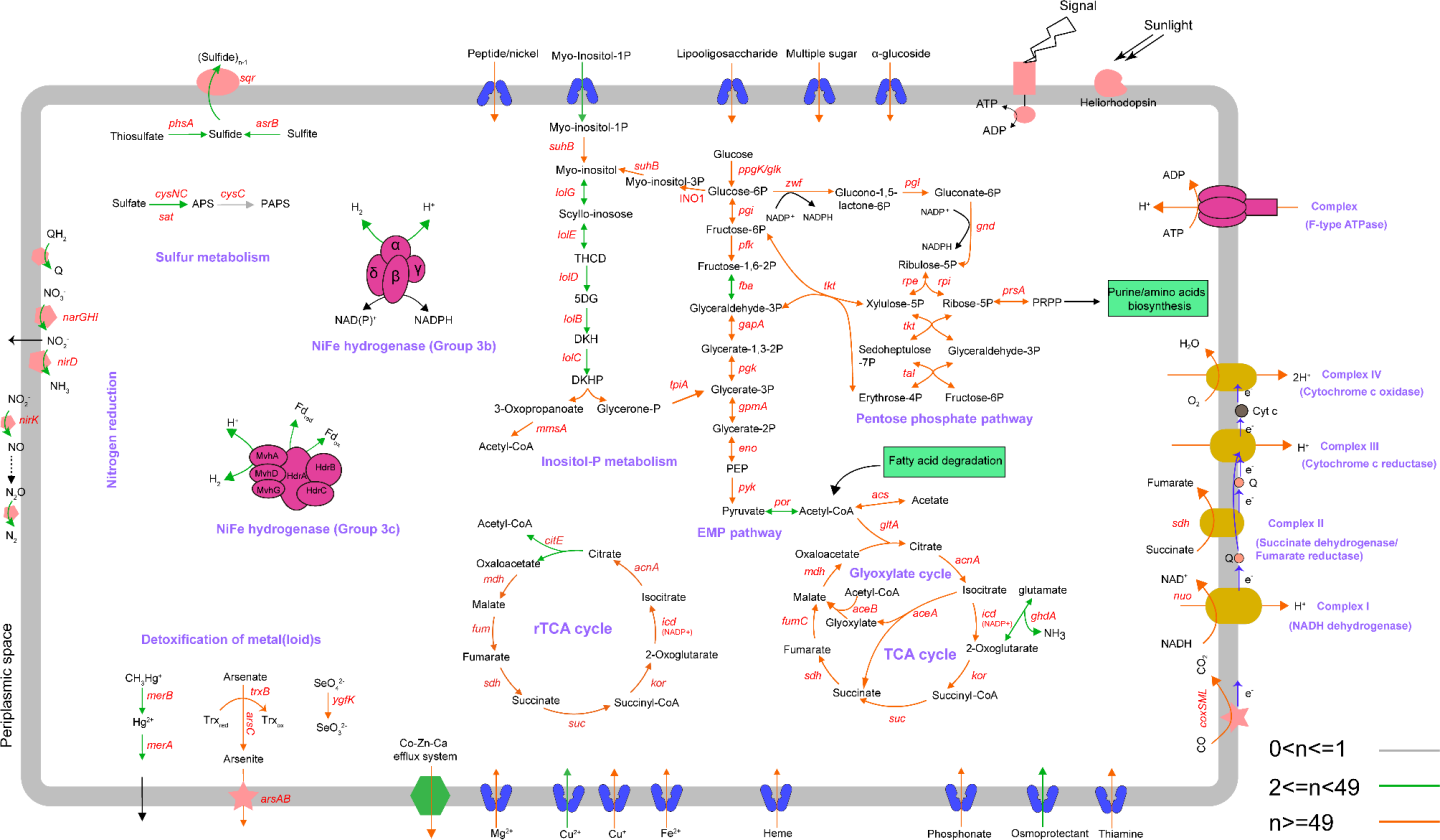
Overall metabolic characteristics of UBA5794 actinobacteria. Genes related to EMP, pentose phosphate pathway, TCA (rTCA) cycle, glyoxylate shunt, inositol-P metabolism, carbon monoxide oxidation, sulfur metabolism, nitrogen reduction, NiFe hydrogenase, metal(loid) detoxification, respiratory chain complexes, and transporters are shown. Different colors of the arrow represent the proportion of genomes containing related genes. A detailed gathering of the pathways and gene copy numbers in each genome is given in Table S5

To learn about the lifestyles of this poorly known order, the overall metabolic characteristics of 98 non-redundant genomes were constructed (Fig. 2; Table S5). Nearly complete glycolysis, also known as EMP (Embden–Meyerhof–Parnas) pathway, was annotated in most UBA5794 genomes. Genes encoding the three key rate-limiting enzymes in glycolysis, namely polyphosphate (polyP) glucokinase (*ppgK*), ATP-dependent phosphofructokinase / diphosphate-dependent phosphofructokinase (*pfk*), and pyruvate kinase (*pyk*), were annotated in 80.61%, 67.35%, and 94.90% of the genomes, respectively, indicating a prevalent heterotroph lifestyle in the UBA5794 clade. Notably, the phosphorylation reaction of glucose in over 80% of UBA5794 can be mediated by polyP glucokinase, in which the phosphate group is provided by polyP rather than ATP. It is known that polyP glucokinase is widespread across prokaryotes and eukaryotes, and that polyP has been recognized as one of the earliest biopolymers [46] and the way of utilizing ATP in the metabolic process probably evolved from polyP-utilization [47]. Thus, the extensive adoption of polyP glucokinase in UBA5794 suggests that these actinobacteria have retained ancient metabolic adaptations. Meanwhile, nearly half of these polyP glucokinase-containing genomes also encode ATP-dependent glucokinase, likely reflects a versatile metabolic strategy that enhance their ability to utilize diverse phosphoryl donors. The evolutionary relationship between these two enzymes falls outside the scope of the current study. However, we maintain that natural selection likely play pivotal roles in their functional maintenance.

More than 75% of UBA5794 genomes contained genes encoding the three key rate-limiting enzymes of TCA cycle, including citrate synthase (*gltA*), isocitrate dehydrogenase (*icd*) (NADP^+^), and 2-oxoglutarate ferredoxin oxidoreductase (*kor*). Earlier studies have shown the NADP dependence of prokaryotic isocitrate dehydrogenase is an ancient adaptation to acetate metabolism, because genomes encoding isocitrate lyase (*aceA*), a key enzyme for growth on acetate through the glyoxylate shunt, always have an *icd* (NADP^+^) simultaneously [48]. A majority of UBA5794 genomes harbored the key genes *aceA* (63.27%) and *aceB* (encoding malate synthase; 57.14%) of the glyoxylate shunt. Considering that the UBA5794 lineages are mainly distributed in relatively oligotrophic environments, where energy-rich compounds such as glucose are scarce, C2 compounds (acetate or acetyl-CoA produced by the oxidation of fatty acids), as a more accessible carbon and energy source [49] in the environments, may be assimilated by most UBA5794. Unconventionally, none of the UBA5794 members possessed 2-oxoglutarate dehydrogenase, instead, most of them contained 2-oxoglutarate ferredoxin oxidoreductase (KOR) subunit alpha (*korA*; 85.71%) and subunit beta (*korB*; 83.67%). As KOR is an oxygen-labile enzyme [50] catalyzing reversible oxidative decarboxylation of α-ketoglutarate to succinyl-CoA, this observation suggests an adaptation of UBA5794 to habitats featured by fluctuations of oxygen concentrations, such as marine sediments, sponges, beach sand, and freshwater sediments [11, 51–53]. However, the homologs of KOR identified in *Mycobacterium tuberculosis* are tolerant to oxygen exposure [54]. Therefore, we deduce that the members of UBA5794 are also able to survive in aerobic environments, and that the prevalent glyoxylate shunt probably endows their oxidative stress tolerance thereby exerting protection on oxygen-labile enzymes in vivo [55]. In many cases, KOR is also identified as a key enzyme in the reductive tricarboxylic acid (rTCA) cycle [56], capable of catalyzing carbon dioxide assimilation reaction under microaerophilic and anaerobic conditions. In addition, the discovery of other key enzymes involved in rTCA pathway, such as citrate lyase subunit beta (*citE*; 32.65%) [57–59] and pyruvate-ferredoxin/flavodoxin oxidoreductase (*por*; 48.98%), further indicated the carbon fixation ability in partial UBA5794 members.

Based on comparison with CAZy database, enzymes belonging to glycoside hydrolase (GH) and polysaccharide lyase (PL) families were observed in UBA5794, with GH109, GH13, and GH1 enriched in over half of the members (Table S6). The predominant GH109 (86.73%) enzymes utilize catalytic mechanism involving NAD^−^ dependent hydrolysis. To validate the specific targeted substrate of GH109 family in UBA5794, all protein sequences hitting to this family were retrieved and then searched against NR database. The blastp results showed that the best hits of most sequences were assigned to Gfo/Idh/MocA family oxidoreductase, which typically works on diverse substrates, such as glucose, *myo*-inositol or *myo*-inositol derivatives, and rhizopine [60]. Furthermore, based on KEGG annotation, genes functioning in *myo*-inositol metabolism were revealed in UBA5794 genomes (Fig. 2; Table S5). The genes encoding the inositol-phosphate transport system (*inoEFG*; 12.24–14.29%) were shown in partial marine sediment lineages. Under the function of *myo*-inositol-1-phosphate synthase (INO1; 77.55%), glucose-6P was transformed into *myo*-inositol-3P, which was further dephosphorized by *myo*-inositol-1(or 4)-monophosphatase (*suhB*; 88.78%) to generate inositol, and the high occurrence of inositol synthesis pathway highlighted the necessity of inositol for UBA5794 members. Besides, a nearly complete inositol dehydrogenase pathway composed of the *iolCGDEB* operon (29.59–35.71%) was mainly distributed in UBA5794 genomes from marine sediments and sponges. In this pathway, inositol was ultimately degraded into glyceraldehyde-3-phosphate, which was further integrated into central metabolism. In addition to being a carbon source, inositol may act as a signal transduction factor in the sponge lineages to regulate sponge-microbe interaction [61]. Additionally, GH13 enzymes targeting starch and other α-glucans and GH1 enzymes degrading cellulose were observed in 77.55% and 56.12% of UBA5794. Other carbohydrate-active enzymes families act on degradation of mannose (GH130), trehalose (GH65), galactose (GH36) and N-acetyl-β-D-glucosamine (GH3) were also present in UBA5794. These results indicate the capacity of UBA5794 to utilize a wide range of carbohydrates.

### Energy metabolism and element cycle

UBA5794 genomes encoded multiple forms of energy conservation (Fig. 2; Table S5). Most of the genomes encoded relatively complete proton-translocating NADH-quinone oxidoreductase subunits (NuoA-NuoN) (Complex I), succinate dehydrogenase complex (Complex II), menaquinol-cytochrome c reductase cytochrome b subunit (Complex III), and cytochrome c oxidase (Complex IV, the terminal enzyme of the aerobic respiratory chain). This result suggests that most UBA5794 lineages retain partial oxidative phosphorylation machinery for energy conservation. Nevertheless, the genes encoding cytochrome c oxidase (*coxABCD*) and cytochrome bd ubiquinol oxidase (*cydAB*) were completely absent in 12.24% of the genomes, most of which (83.33%) were derived from marine sediments. Integrating additional genomic evidence—notably the absence of oxygen-detoxifying enzymes (e.g., catalase and superoxide dismutase) —and the predominant isolation of these lineages from deep-sea sediment environments, we propose that they are likely physiologically specialized for anaerobic habitats, with their metabolic architecture reflecting adaptation to oxygen-limited conditions. Carbon monoxide is an alternative energy source for marine microbes [49], and three subunits of aerobic carbon monoxide dehydrogenase (CoxLMS) were found in over 70% of the genomes.

Molecular hydrogen can be utilized by prokaryotes across aquatic, terrestrial, and even host-associated ecosystems under aerobic or anaerobic conditions [62, 63]. After re-classification based on hydrogenase classifier HydDB, [NiFe] hydrogenases groups 3b and 3c, namely sulfhydrogenase (HydABDG; 2.04-35.71%) and F420-reducing hydrogenase (MvhADG; 8.16-10.20%), respectively, were detected in some UBA5794, with group 3b being the dominant type. Notably, the genes for sulfhydrogenase always co-occurred with the gene for anaerobic sulfite reductase subunit B (*asrB*; 24.49%), whereas the *hydG* was only found in two UBA5794 genomes. In the hydrogenase of *Pyrococcus furiosus*, the HydG subunit is extensively homologous to AsrB, probably endowing sulfhydrogenase with specialized sulfur reducing activity [64]. Therefore, we speculated that in the sulfhydrogenase gene cluster of UBA5794 genomes, *asrB* may serve as an equivalent of *hydG* to dispose excess reductant using sulfites as electron acceptors. These hydrogen-consuming sulfate reducers have potentials to use sulfite as the electron acceptor in couple with H_2_ oxidation for energy-producing process [65]. However, hydrogenase was completely absent in UBA5794 genomes derived from sponges.

Additionally, UBA5794 appeared to possess other pathways for sulfur metabolism, although no related genes were found in the genomes of the sponge lineages. The gene encoding sulfide-quinone reductase (SQR) was present in 56.12% of the UBA5794 genomes, indicating that oxidation of sulfides may serve as an available energy source for most free-living UBA5794 [66]. High concentrations of sulfides readily accumulate in certain environments, such as hydrothermal vents, coastal mudflats, and river, and are typically produced by sulfate-reducing bacteria [66–68]. In addition to providing energy for physiological metabolic activity of chemolithotrophic bacteria, sulfur oxidation also plays an important role in relieving the toxicity of sulfides to cells [69]. For assimilatory sulfate reduction, genes of *cys* family were detected in several UBA5794 genomes, but none of them contained a complete *cys* gene cluster.

Genome annotation results also revealed the potential for dissimilatory nitrate reduction in UBA5794, as exemplified by genes encoding membrane-bound nitrate reductase (*narGHI*; 14.29-17.35%), nitrite reductase small subunit (NirD; 15.31%) participating in the subsequent dissimilatory nitrate reduction to ammonium (DNRA), and nitrite reductase (NO-forming) (*nirK*; 32.65%) and nitrous oxide reductase (*nosZ*; 8.16%) in the process of denitrification. As previously described, DNRA and denitrification are limited to extremely hypoxic oxygen minimum zones (OMZs) [70] and marine sediments (<∼5 µM O_2_). Thus, some UBA5794 may be able to conserve energy through nitrate respiration. Similar to the results of sulfur cycle, no genes involved in nitrogen cycle were found in sponge-derived UBA5794 genomes. Functional redundancy of nitrogen and sulfur metabolic pathways has been revealed in sponge-associated microbial communities [12], so we speculated that the missing functions in sponge-derived UBA5794 might be compensated by other symbiotic microorganisms in the sponges. However, this hypothesis requires future experimental validation, such as metabolite tracing or co-culture assays, to confirm the proposed metabolic interdependencies.

### Reduction and detoxification of heavy metal(loid)s

Moreover, the UBA5794 members harbor genomic components in charge of the transformation of heavy metals or metalloids in the environments for energy conservation and detoxification (Fig. 2; Table S5). The prevalence of the *arsRABC* operon involved in arsenate reduction in UBA5794 suggests their capability of driving biogeochemical arsenate cycle. Arsenate reductase encoded by *arsC* was annotated in 72.45% of the genomes, catalyzing the transformation of arsenate (As[V]) to arsenite (As[III]) by intramolecular thiol-disulfide cascade using thioredoxin (Trx) as a reductant in redox reactions [71]. The ability of subsequent methylation of arsenite was also shown in UBA5794 genomes, with 58.16% of them possessing *arsM* that encodes arsenite S-adenosyl-methionine methyltransferase, resulting in volatile methylated arsine species such as dimethylarsine and trimethylarsine. Therefore, arsenic methylation mediated by the UBA5794 lineages may play an import role in bioremediation of As-contaminated environments [72, 73]. The *mer* operon responsible for the biological methylmercury demethylation (*merB*, alkylmercury lyase) and mercury (Hg[+ II]) reduction detoxification (*merA*, mercuric reductase) processes was mainly identified in UBA5794 genomes derived from marine sediments. The capacity for tolerance and degradation of methylmercury may confer survival advantage to UBA5794 marine sediment lineages against the widespread distribution of toxic mercury in the ocean [74]. Furthermore, based on the identification of genes encoding YgfKMN selenate-reducing complex in UBA5794 lineages, with *ygfK* (putative selenate reductase) found in 53.06% of the genomes, selenate appears to be an available electron acceptor in anaerobic respiratory processes of UBA5794 [75]. Presumably, downstream selenite reduction may be mediated by glutathione reductase or thioredoxin reductase encoded by most UBA5794 genomes, or through non-enzymic reactions where selenite reacts with thiol-containing proteins or reduced glutathione, finally resulting in the end-product elemental selenium [76, 77].

### Separation between sponge-associated and free-living UBA5794 groups and their habitat adaptation

**Fig. 3:**
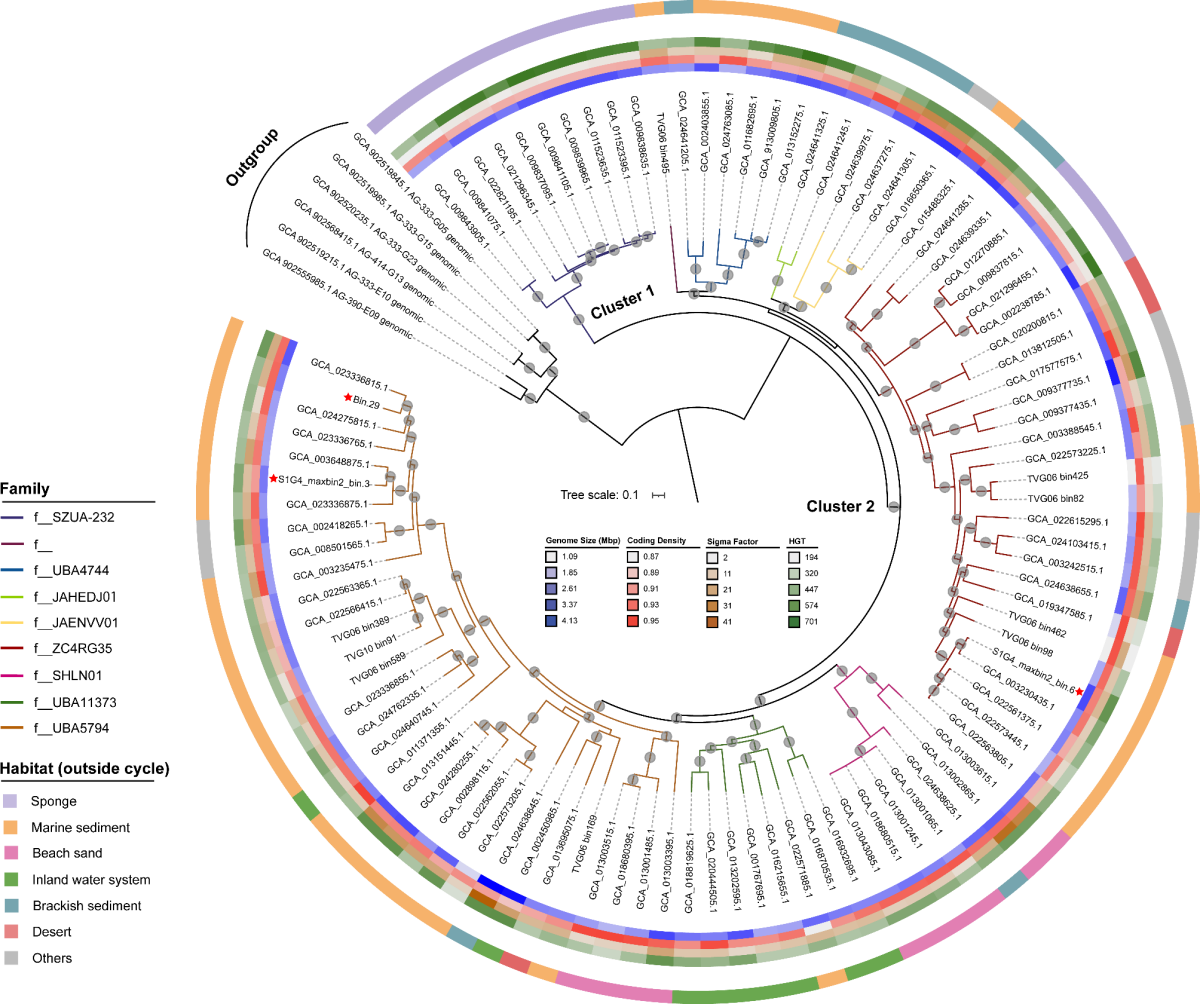
Genome-wide phylogenetic analysis of UBA5794 lineages. A total of 98 non-redundant UBA5794 genomes were collected from the GTDB database, Southwest Indian Ocean sediments dataset [31], and this study (marked with red stars). The tree was constructed with IQ-tree (v2.1.2) based on the concatenated alignment of 291 OGs shared between UBA5794 and “*Ca*. Actinomarinales”. Branches were colored according to the classification at the family level. The connected circle layers represent estimated genome size, coding density, sigma factor numbers, and HGT numbers from inside to outside. Colors of the outermost cycle reflect the habitat sources. Bootstrap values over 90% of 1,000 resamples are shown as grey dots. The two clusters of UBA5794 are labelled with “Cluster 1” and “Cluster 2”

**Fig. 4:**
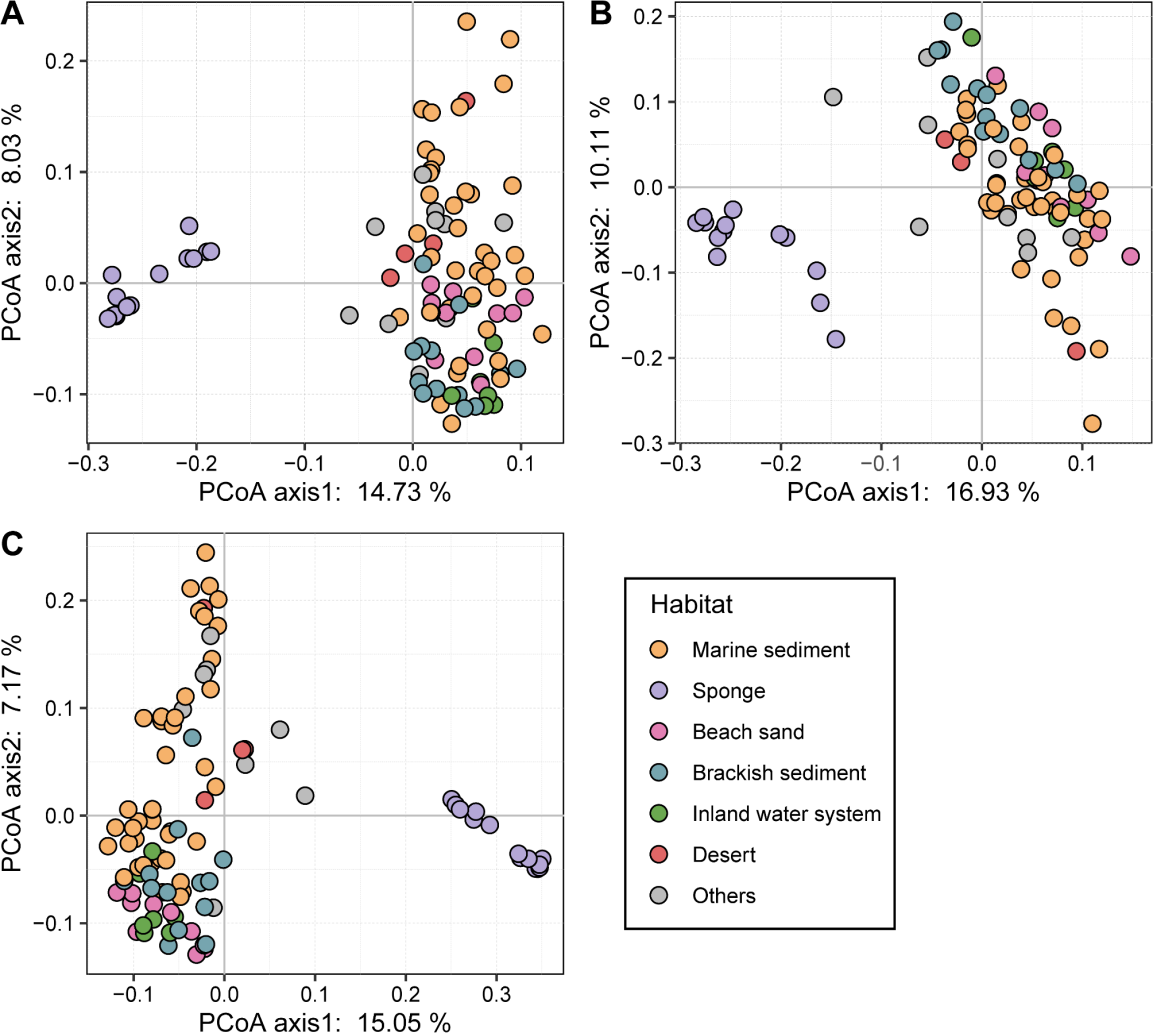
Ordination of functional gene contents of 98 non-redundant UBA5794 genomes derived from different habitats. PCoA analysis of (**A**) KO, (**B**) PFAM, and (**C**) OG matrices was performed based on Bray-Curtis dissimilarities

The phylogenomic tree of UBA5794 based on concatenation of shared OGs could be divided into two highly supported clusters using the sister order “*Ca*. Actinomarinales” as outgroup (Fig. 3). Cluster 1 was exclusively composed of sponge-associated members, whilst Cluster 2 included a variety of members from diverse habitats (Fig. 3). To investigate the functional differentiation in UBA5794 inhabiting different habitats, the CDS sequences of UBA5794 genomes were clustered by PCoA analysis based on the Bray-Curtis distance of their KOs, PFAMs, and OGs (Table S7-S9). No matter what method was used, all sponge-associated members were clustered together and clearly distinguished from those of other habitats (Fig. 4), suggesting that the symbiont lifestyle has largely shaped the functions. In light of these findings, we divided all UBA5794 into sponge-associated group and free-living group.

**Fig. 5:**
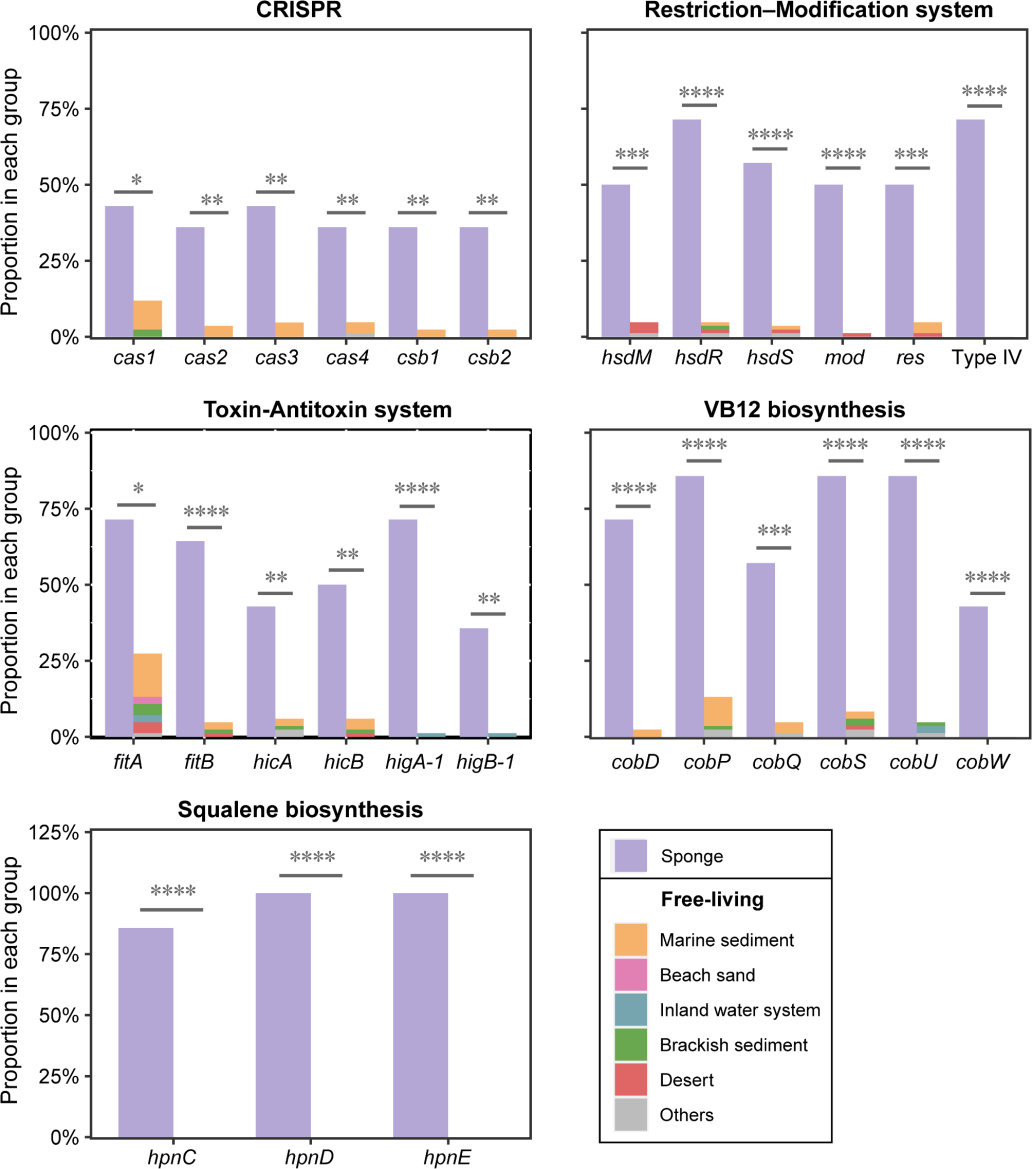
Representative genes/domains enriched in the genomes of sponge-associated UBA5794. Bar plots show the differences in the proportion of de-redundant genomes containing related genes within the two different habitat groups. The sponge group is arranged separately as the left column, and the free-living group composed of members dwelling from different open environments is stacked to form the right column. The different numbers of stars above the column indicate significant difference to varying degrees: *, *P* < 0.05; **, *P* < 0.01; ***, *P* < 0.001; ****, *P* < 0.0001

To retrieve the underlying genes associated with habitat adaptation, we performed gene enrichment analysis with Scoary [42]. In total, 298 KOs, 295 PFAMs, and 997 OGs (among which 761 were annotated by DIAMOND) were significantly correlated to sponge-associated habitats; whilst 236 KOs, 294 PFAMs and 516 OGs (493 annotated by DIAMOND) were significantly correlated to open environments (Table S10-S12). Sponges are hosts to a considerably variety of mobile genetic elements, such as plasmids, transposable elements, and viruses, and therefore, the sponge symbiotic microorganisms likely require multiple defense mechanisms to resist foreign infections [78, 79]. We detected that immune repertoire related genes were significantly enriched in the sponge-associated UBA5794 genomes, including those for type I (*hsdM*, *hsdR*, *hsdS*), III (*mod*, *res*), and IV restriction–modification systems, CRISPR-associated proteins (Cas1, 2, 3, 4 and Csb1, 2), and type II toxin-antitoxin systems (*fitA*/*fitB*, *hicA*/*hicB*, *higA*-1/*higB*-1) (Fig. 5). These defense systems may be critical for symbiotic UBA5794 in sponges, allowing them to cope with adverse conditions of extreme exposure to potentially harmful foreign microorganisms or viruses, thereby colonizing and persisting in the host. Previous genomic studies on sponge symbionts have shown their potential for vitamin biosynthesis, including thiamine (VB1), riboflavin (VB2), pyridoxine (VB6), biotin (VB7), and cobalamin (VB12) [79, 80]. It was also observed in our study that genes involved in the synthesis of VB12 (*cobQDPSUW*) were enriched in sponge-associated UBA5794 lineages (Fig. 5). These symbionts may serve as an alternative source of vitamins for their sponge hosts, thus conferring them a fitness advantage that facilitates a stable symbiotic relationship with sponge tissues, as suggested by previous findings [80]. Moreover, we found that the gene cluster for squalene biosynthesis, which consists of three highly conserved genes—*hpnC*, *hpnD*, and *hpnE* [81]—was restricted to the sponge-associated group (Fig. 5; Fig. S5). Squalene is a natural isoprenoid hydrocarbon and a crucial precursor for the biosynthesis of steroids and terpenoids [82, 83], and terpenoids are commonly found in marine sponges [84]. As secondary metabolites found in sponges are often related to the microorganisms they host [79], the UBA5794 sponge lineages may supply squalene as a precursor to their hosts for further biosynthesis of complex terpenoid compounds.

**Fig. 6:**
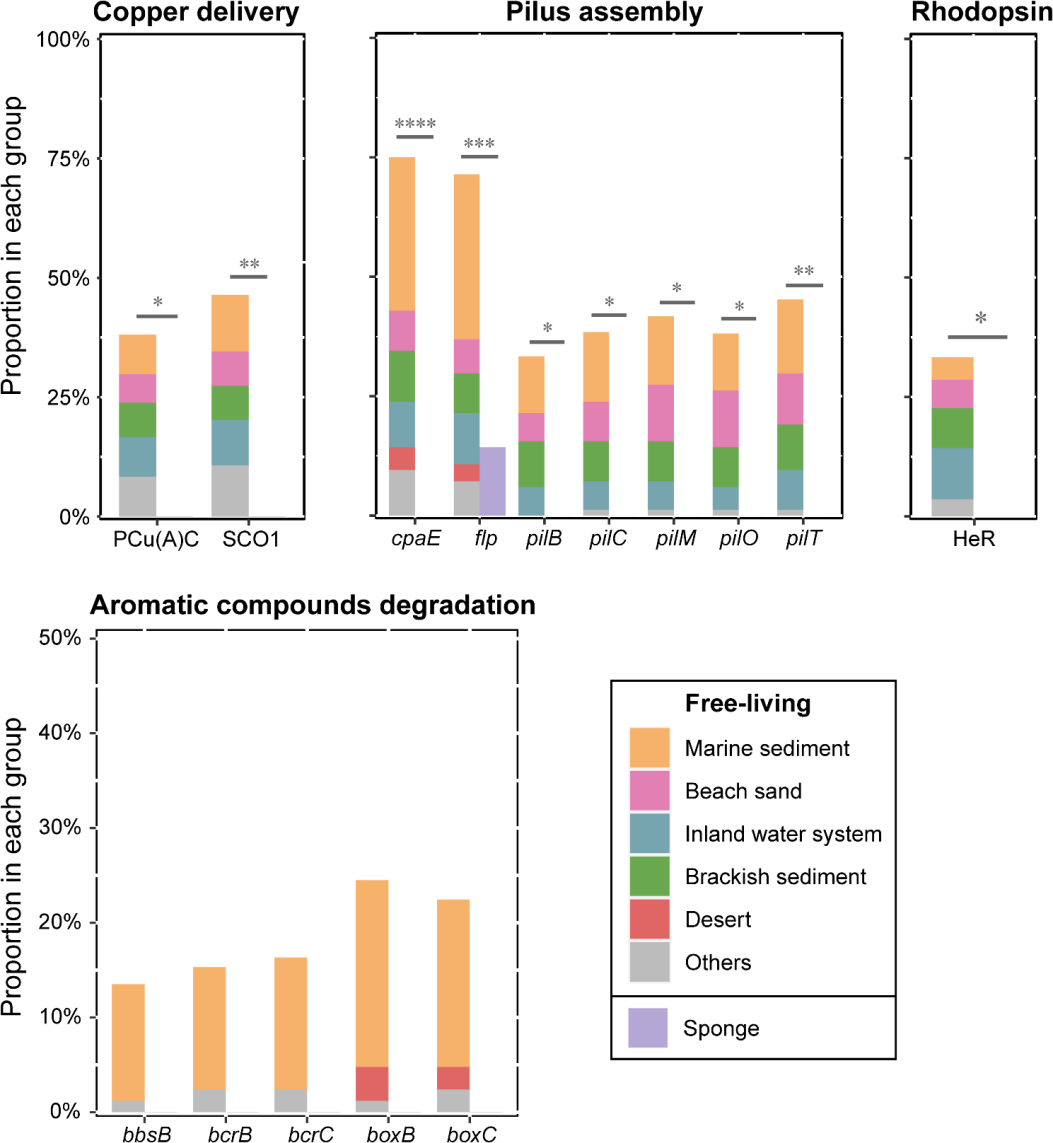
Representative genes/domains enriched in the UBA5794 genomes derived from open environments. Bar plots show the differences in the proportion of de-redundant genomes containing related genes within the two different habitat groups. The group of different open environment sources is stacked to form the left column, and the group of sponge sources is arranged separately as the right column. The different numbers of stars above the column indicate significant difference to varying degrees: *, *P* < 0.05; **, *P* < 0.01; ***, *P* < 0.001; ****, *P* < 0.0001

Copper ions are the essential catalytic cofactor for many important enzymes, required for fundamental intracellular processes such as respiration, antioxidant defense, and pigment formation [85, 86]. However, according to existing KEGG and Pfam annotation results, SCO1, which is responsible for the correct assembly of copper with cytochrome C oxidase, and copper chaperone PCu(A)C are completely missing in the sponge group (Fig. 6). Referring to the previous work [87, 88], we speculate that P-type Cu^+^ transporter encoded by sponge-associated UBA5794 genomes may be involved in the protein metalation, translocating Cu^+^ to copper-dependent periplasm enzymes, while also conferring Cu^2+^ tolerance to the sponge group. Nevertheless, some hypothetical proteins in the sponge group may also engage in the mediation of copper trafficking, which remains a need for future investigations.

Although free-living UBA5794 actinobacteria are distributed among diverse habitats, they still share a series of genomic traits. Genes involved in both Type IVa (*pilABCEMOTY1*) and Type IVc (Tad; *capBCEF*) of Type IV pili system assembly [89] were revealed in free-living UBA5794 genomes from different habitats; in contrast, the Type IVa genes and *capE* were absent in sponge genomes (Fig. 6). Type IV pili systems are widespread in major phyla of prokaryotes, which may endow free-living UBA5794 with functions of twitching motility, forming microcolonies and biofilms, signal sensing, and DNA uptake [90]. And flagellar assembly and motility proteins were only found in several members from marine and brackish sediments (Table S5), which may enhance their athletic ability to migrate towards more desirable niches. In addition, the free-living genomes harbored diverse genes involved in the transformation of inorganic matter, such as sulfite, nitrite, and hydrogen (Fig. 2), as described above. Although these metabolic pathways were virtually absent in sponge-associated UBA5794, genes encoding transport systems and metabolism of organosulfur compound taurine were enriched in their genomes (Table S10), in line with the fact that sponge symbionts have widespread potential to use host-derived organic metabolic products [79].

Microbial rhodopsin photosystems are considered the most universal strategy to convert light into energy and signals [91]. Pfam annotation revealed the heliorhodopsin (HeR) domain in UBA5794, mostly present in those derived from beach sand, brackish sediments, and inland water, where cells are under intensive exposure to sunlight (Fig. 6). Phylogenetic analysis of HeR sequences of UBA5794 showed that they were largely assigned to the *Actinobacteria*-dominant clade, close to homologs from ubiquitous freshwater actinobacterial “*Ca.* Nanopelagicus abundans” and “*Ca.* Planktophila limnetica” (Fig. S6). Only two UBA5794 HeR sequences were clustered with the “*Ca*. Actinomarinales” sequences in the other clade with higher taxonomic diversity. Moreover, we noticed that genes encoding histidine kinase-, DNA gyrase B-, and HSP90-like ATPase (HATPase) domains were present in the vicinity of HeR genes (Fig. S7), in many cases together with genes coding for transcriptional regulation (e.g., LuxR family, GNAT family, and Sigma70), transporter (e.g., ABC_tran, MFS, Ion_trans), and protein hydrolysis (e.g., hydrolase_4, peptidase and amino_oxidase) (Fig. S7). Besides, the genes of protein domains associated with DNA repair were also found to be adjacent to HeRs, such as DNA photolyase and Radical_SAM (Fig. S7). This observation suggests that, in HeRs-containing UBA5794, light signal is likely transmitted through their sensory HeRs linked to signal transduction systems, triggering the increase in downstream transportation and utilization of available resources as well as response to photo-induced damage [92].

Some UBA5794 members from marine sediments possessed the potential for degrading aromatic compounds, as they contained genes encoding benzoylsuccinyl-CoA thiolase BbsA subunit (*bbsA*) and benzoyl-CoA reductase (*bcrBC*), which are involved in anaerobic degradation of toluene, and benzoyl-CoA 2,3-epoxidase (*boxBC*) involved in the aerobic benzoate metabolism (Fig. 6). Hydrocarbons released during oil spills in marine sediments have a broad range of toxicity on marine organisms, which may force marine benthic UBA5794 to develop effective survival strategies coping with these toxic compounds.

### Lifestyle and HGT shaping habitat adaptation

There is no significant difference in genome size between sponge-associated and free-living UBA5794 groups (t-test, *P* > 0.05; Fig. S4A). However, compared to the free-living group, the sponge group has significantly lower genome coding density (t-test, *P* < 0.01; Fig. S4B) and smaller number of sigma factors (Wilcoxon test, *P* < 0.01; Fig. S4C). These features are consistent with the symbiotic lifestyle. Benefiting from constant association with their sponge hosts and other microorganisms inhabiting the sponges, some of the functional genes of the sponge group may be non-essential and gradually turn into pseudogenes with the accumulation of mutations, probably related to the initial loss of sigma factors through genetic drift [93, 94].

HGT is a crucial mechanism in the evolution and adaptation of microbial genomes, considerably affects patterns of genome variation [95]. We therefore detected and compared the distribution of HGT events among UBA5794 genomes. The number of predicted HGTs in each UBA5794 genome ranges from 194 to 701 (Fig. 3; Table S13). Comparisons of HGT numbers (Wilcoxon test, *P* < 0.05; Fig. S8A) and HGT numbers normalized by genome size (Wilcoxon test, *P* < 0.05; Fig. S8B) both showed that the overall HGT frequency in the sponge genomes was significantly higher than that in the free-living genomes. Then, all the HGT-related genes were annotated using KEGG and Pfam databases (Table S14-15), and PcoA analysis was conducted based on the obtained homologous KO and PFAM matrices. The clustering results based on HGT relevant genes still indicated that the sponge group was obviously separate from free-living group (Fig. S9). Further analysis of the annotation results of HGT genes revealed that the sponge-associated genes involved in defense systems (CRISPR, restriction–modification, and toxin-antitoxin system) and vitamin synthesis were probably derived from HGT events, likely reflecting adaptations to host immune pressures and symbiotic nutrient provisioning. As for the free-living group, genes encoding nitrite reductase (*nirK*), sulfide: quinone oxidoreductase (*sqr*), protein SCO1, benzoyl-CoA 2,3-epoxidase subunit B (*bcrB*), and benzoyl-CoA-dihydrodiol lyase (*bcrC*) were potentially acquired from phylogenetically distant donors. These genes may provide free-living UBA5794 with additional energy acquisition strategies and enhanced adaptability in toxic environments. Taken together, while sponge microbiomes may inherently promote HGT due to their densely colonized nature, the functional bias of transferred genes—towards host interaction in symbionts and energy conservation in free-living lineages—strongly implicates HGT as a driver of habitat adaptation. This functional partitioning underscore the selective pressure exerted by distinct ecological niches on genome evolution.

### Ancestral habitat reconstruction

**Fig. 7:**
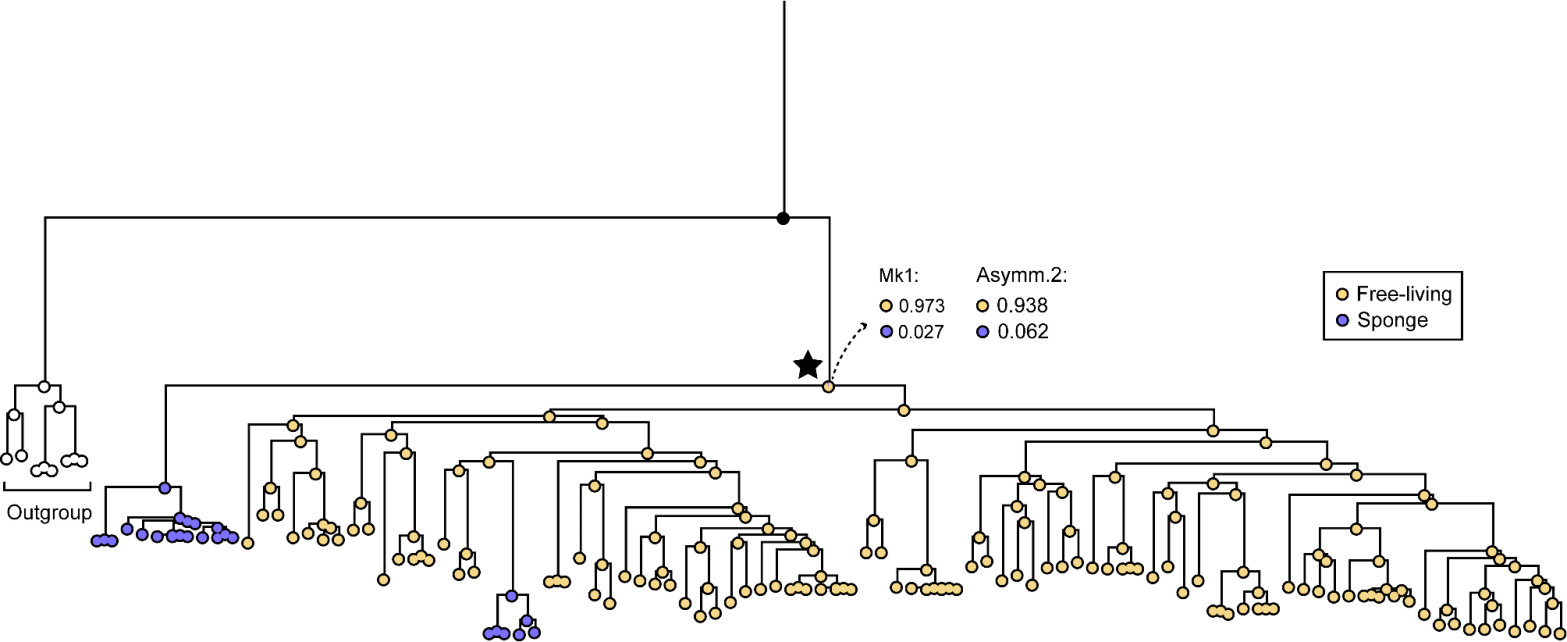
Ancestral state reconstruction of habitats. Marginal probabilities were calculated based on the habitat states using the Mk1 and Asymm.2 probability models of ML ancestral states. Leaf nodes of the phylogenetic tree based on 291 OGs are colored by the habitat sources as follows: purple, sponge; yellow, free-living environment, and the colors of internal nodes are displayed in proportion to probability. The MRCA node of UBA5794 is marked with a solid star. Probability of the MRCA is shown in the top right of corresponding nodes

To infer the evolutionary history of habitat transitions across the UBA5794 phylogeny, we first reconstructed the ancestral habitat state of this order based on the phylogenomic tree and the separation between sponge-associated and free-living groups. By using ML analysis with Mk1 and Asymm.2 models in Mesquite, we predict that the most recent common ancestor (MRCA) of UBA5794 evolved in a free-living environment (Mk1 probability = 0.973; Asymm.2 probability = 0.938) (Fig. 7). During the successful colonization of some ancient UBA5794 lineages into sponge hosts, a significant number of HGT events render them a highly distinct UBA5794 lineage, Cluster 1 (Fig. 3), capable of adapting to the symbiont environments. However, there exists another smaller sponge-associated UBA5794 lineage, affiliated in the same family with free-living lineages (f_ ZC4RG35) in Cluster 2, showing higher phylogenetic conservation than Cluster 1 (Fig. 3). It is likely that a few sponge-associated members are still undergoing an evolutionary transition from free-living to sponge-associated habitats, although their functions have been largely shaped by the symbiont lifestyle (Fig. 4).

Interestingly, members of the “others”, which are mainly distributed in trackless habitats such as Moville Cave, permafrost, desert, and dinosaur fossils, were clustered with the marine sediment lineages (Fig. 3). Considering the diverse habitats of UBA5794 and using the Mk1 probability model of ML ancestral states, it is possible, although with inherent uncertainty, that the MRCA might have evolved from marine sediments (probability = 0.607; Fig. S10). This result implies that the “others” might originate from marine sediments. It is speculated that under the strong external force of plate tectonics, the “others” might be geographically isolated from the marine sediment lineages. However, due to their distribution in less disturbed habitats, their evolutionary rates may be relatively slow, allowing for the preservation of primitive lineage characteristics.

## Conclusions

UBA5794 actinobacteria are abundant in marine but also distributed in some terrestrial habitats. Our results demonstrate that UBA5794 should be considered as an independent order in parallel with previously described “*Ca*. Actinomarinales” within the class *Acidimicrobiia*. The highly flexible metabolism and energy conservation strategies of UBA5794 enable them to thrive in diverse habitats. This order performs anaerobic or microaerophilic respiration in adaptation to widespread hypoxic environments. UBA5794 members can utilize a wide range of carbon sources from CO_2_ to inositol, starch and cellulose, with H_2_, CO or sulfide as an electron donator. Moreover, many UBA5794 members have evolved several metal resistance genes to alleviate the stress from the toxicity of heavy metal(loid). Our results also indicate that lifestyle and horizontal gene transfer play a major role in driving the divergence between the sponge-associated and the free-living UBA5794 groups. The sponge-associated members of UBA5794 have a rich immune repertoire and may supply cobalamin and squalene to their hosts for a symbiotic lifestyle, whilst the free-living members exhibit ecological flexibility by employing strategies such as high motility and the ability to transform inorganic matter, produce HeRs and degrade toxic monoaromatics. Finally, we show that the ancestor of UBA5794 likely originated from free-living habitats and subsequently migrated to sponge hosts.

## Electronic supplementary material

Below is the link to the electronic supplementary material.

**Supplementary Material 1: Figure S1.** ML Phylogenetic tree of all 16S rRNA genes affiliated with the order “*Ca*. Actinomarinales” in the Silva 138.1 database. The tree was constructed using FastTree (v2.1.3) (GTR model). Yellow range reflects the family “*Ca*. Actinomarinaceae”, and red range reflects the family “uncultured”. Branches of our OTUs were highlighted in red. Bootstrap values over 90% were indicated as dots at branch points.

**Supplementary Material 2: Figure S2.** Phylogenetic placement of thirteen main OTUs and 16S rRNA gene of UBA5794 MAGs recovered from this study. The well-supported branches clustered by sequences retrieved from MAGs and OTU were shaded in gradient red. Bootstrap values are texted near the nodes.

**Supplementary Material 3: Figure S3.** Genome-wide phylogenetic analysis of class *Acidimicrobiia* based on the 120 bacterial concatenated marker proteins identified by GTDB-Tk. All the orders of *Acidimicrobiia* by the GTDB taxonomy are included in this ML phylogenomic tree, with outgroups of nearby actinobacterial classes (*Actinomycetes*, *Coriabacteriia*, and *Thermoleophilia*). Bootstrap values were indicated as dots at branch points.

**Supplementary Material 4: Figure S4.** Statistical comparison of genomic features between free-living and sponge groups. T test was used to determine the difference in genome size (A) and coding density (B) between the two groups. (C) Wilcoxon test was used to determine the difference in the number of sigma factors between the two groups.

**Supplementary Material 5: Figure S5.** Squalene biosynthesis gene clusters in UBA5794 genomes from the sponge lineages.

**Supplementary Material 6: Figure S6.** ML phylogenetic tree of the heliorhodopsin (HeR) protein. HeR sequences from UBA5794 and “*Ca*. Actinomarinales” genomes were colored in red and orange, respectively. Bootstrap values over 50% were marked at branch nodes.

**Supplementary Material 7: Figure S7.** Organizations of UBA5794 genomic regions including HeR genes and related genes in the neighborhood. Colors in the left column indicate different habitat sources as follows: orange, marine sediments; pink, beach sand; green, inland water systems; blue, brackish sediments. HeR genes were highlighted in red, and the genes coding for protein domains in the vicinity are colored in accordance with functional classification.

**Supplementary Material 8: Figure S8.** Statistical comparison of predicted HGT numbers between free-living and sponge derived genomes. Wilcoxon test was used to determine the difference in HGT numbers (A) and standardized HGT numbers (the rate of predicted HGT to genome size) (B) between the two groups.

**Supplementary Material 9: Figure S9.** Ordination of functional annotation of identified HGT genes in 98 non-redundant UBA5794 genomes derived from different habitats. PCoA analysis of (A) KO and (B) PFAM matrices was performed based on Bray-Curtis dissimilarities.

**Supplementary Material 10: Figure S10.** Ancestral state reconstruction of habitats. Marginal probabilities were calculated based on the habitat states (marine sediment, sponge, beach sand, inland water system, brackish sediment, desert and others) using the Mk1 probability model of likelihood ancestral states. Leaf nodes of the phylogenetic tree based on 291 OGs are colored by the habitat sources, and the colors of internal nodes are displayed in proportion to probability. The MRCA node of UBA5794 is marked with a solid star. Probabilities of the MRCA and the ancestor of cluster 2 are shown in the top right of corresponding nodes.

**Supplementary Material 11: Table S1.** Relative abundance of non-singleton OTUs assigned to *Actinomarinales* at 97% sequence similarity in sample N04.

**Supplementary Material 12: Table S2.** General genomic and assembly information.

**Supplementary Material 13: Table S3.** Characteristics of the metagenome-assembled genomes in enriched N04.

**Supplementary Material 14: Table S4.** Information about refined UBA5794 genomes from this study and additional UBA5794 genomes collected from GTDB database and our previous study.

**Supplementary Material 15: Table S5.** List of KOs involved in the main pathways as shown in Fig. 2.

**Supplementary Material 16: Table S6.** CAZy-based annotation of 98 non-redundant UBA5794 genomes.

**Supplementary Material 17: Table S7.** KEGG-based annotation of 98 non-redundant UBA5794 genomes.

**Supplementary Material 18: Table S8.** Pfam-based annotation of 98 non-redundant UBA5794 genomes.

**Supplementary Material 19: Table S9.** Orthogroups shared by UBA5794 and “*Ca*. Actinomarinales” genomes.

**Supplementary Material 20: Table S10.** Habitat-associated KOs across genomes of sponge symbiont and free-living members.

**Supplementary Material 21: Table S11.** Habitat-associated PFAMs across genomes of sponge symbiont and free-living members.

**Supplementary Material 22: Table S12.** Habitat-associated Orthogroups across genomes of sponge symbiont and free-living members.

**Supplementary Material 23: Table S13.** Horizontal gene transfer (HGT) events identified in 98 non-redundant UBA5794 genomes.

**Supplementary Material 24: Table S14.** KEGG-based annotation of HGT genes identified in 98 non-redundant UBA5794 genomes.

**Supplementary Material 25: Table S15.** Pfam-based annotation of HGT genes identified in 98 non-redundant UBA5794 genomes.

## Data Availability

All data for this study can be found under the NCBI Bioproject ID PRJNA1141458. The metagenomic raw data for enriched N04 sample (N04SN) are available at NCBI under the accession number of SAMN43058488 and the MAGs can be found under the accession numbers of SAMN42893693 to SAMN42893727 and SAMN42893734 to SAMN42893736.
