# Supplementary figures and images for: Diverse lifestyles and adaptive evolution of uncultured UBA5794 actinobacteria, a sister order of “Candidatus actinomarinales”
Source: Environ Microbiome. 2025 Apr 19;20:39. doi: 10.1186/s40793-025-00701-w (PMC12008989; doi:10.1186/s40793-025-00701-w)

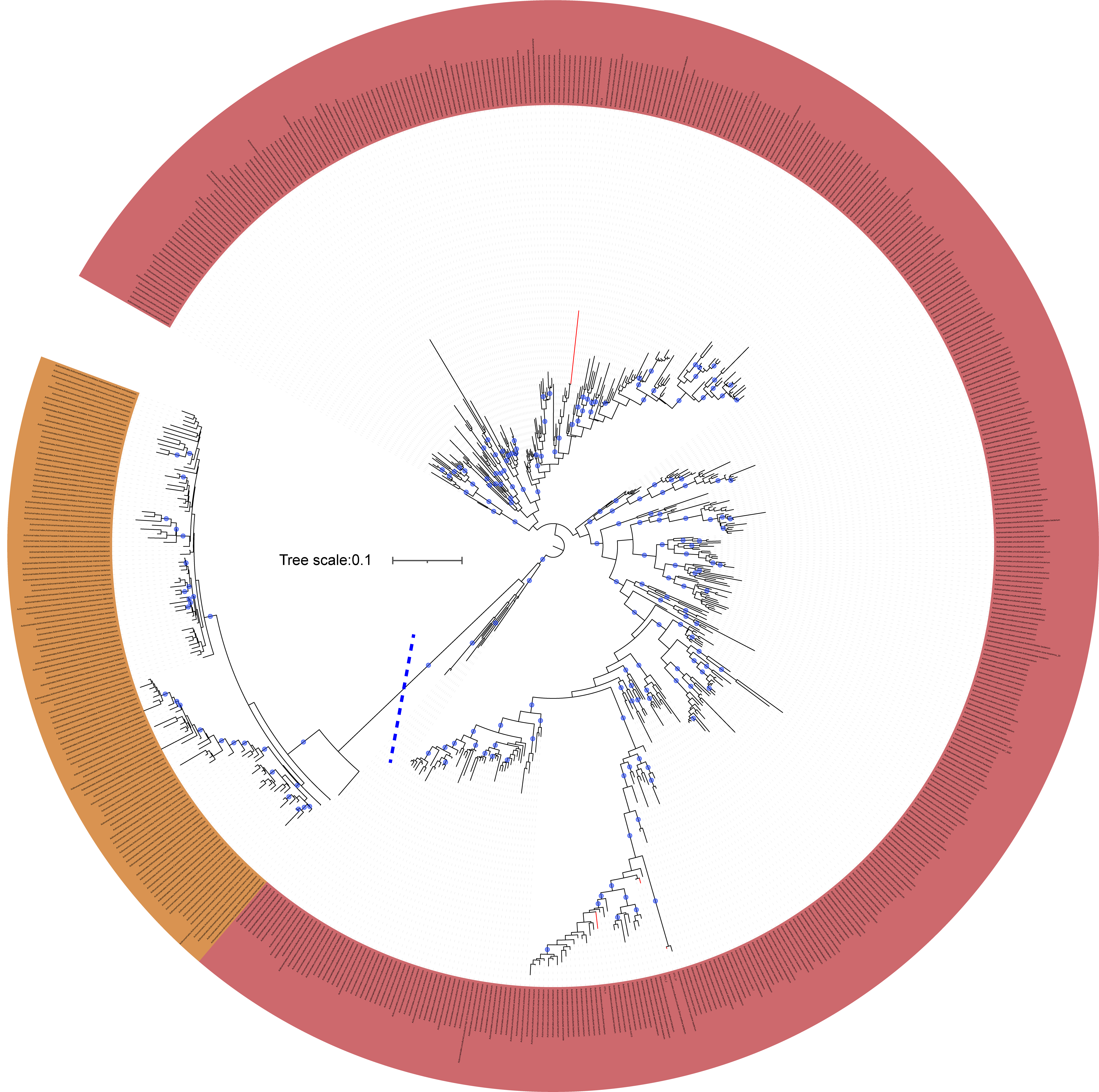

Supplement: Supplementary file 1 — Supplementary Material 1: Figure S1. ML Phylogenetic tree of all 16S rRNA genes affiliated with the order “Ca. Actinomarinales” in the Silva 138.1 database. The tree was constructed using FastTree (v2.1.3) (GTR model). Yellow range reflects the family “Ca. Actinomarinaceae”, and red range reflects the family “uncultured”. Branches of our OTUs were highlighted in red. Bootstrap values over 90% were indicated as dots at branch points. [file 40793_2025_701_MOESM1_ESM.tif]

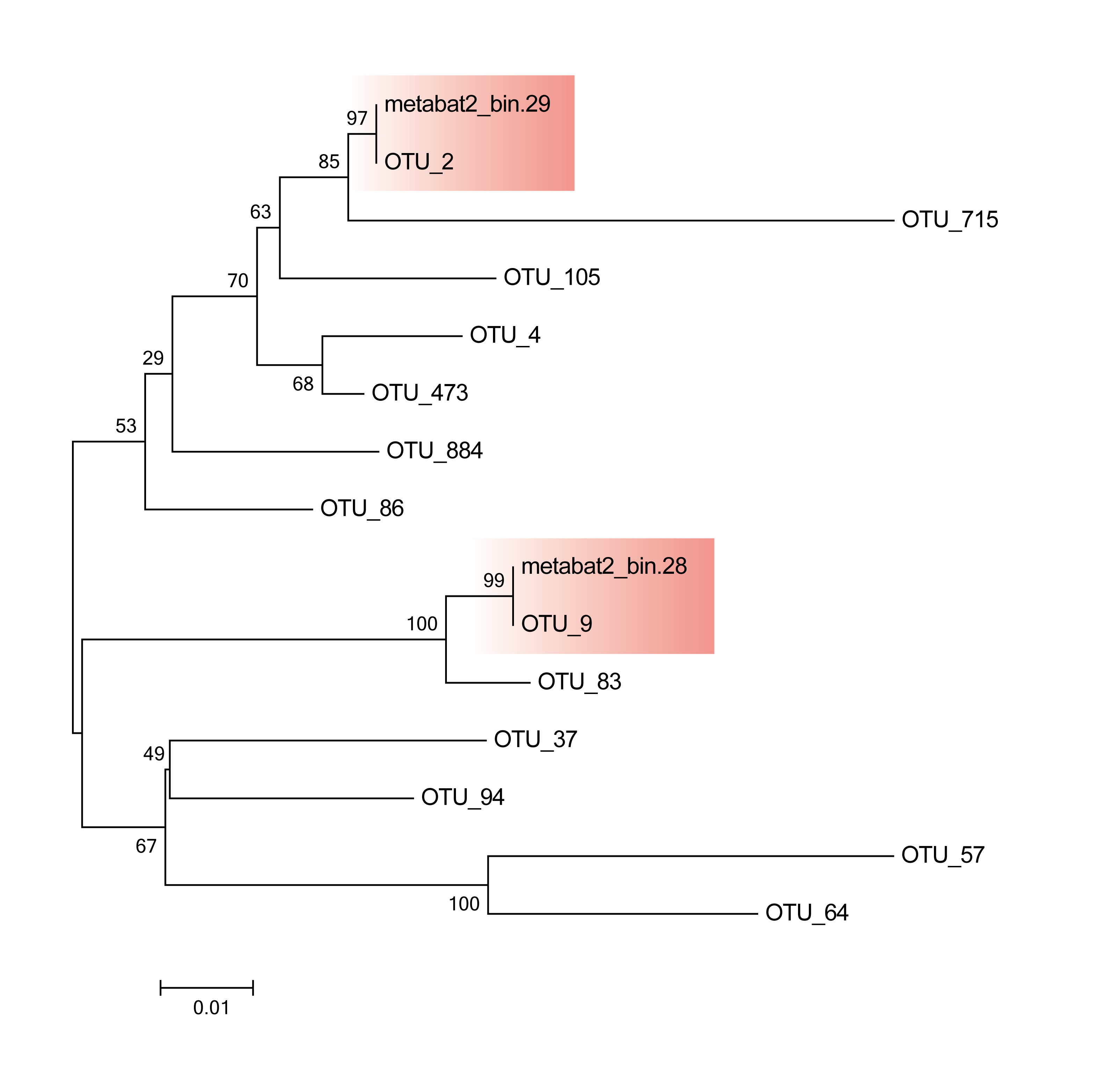

Supplement: Supplementary file 2 — Supplementary Material 2: Figure S2. Phylogenetic placement of thirteen main OTUs and 16S rRNA gene of UBA5794 MAGs recovered from this study. The well-supported branches clustered by sequences retrieved from MAGs and OTU were shaded in gradient red. Bootstrap values are texted near the nodes. [file 40793_2025_701_MOESM2_ESM.tif]

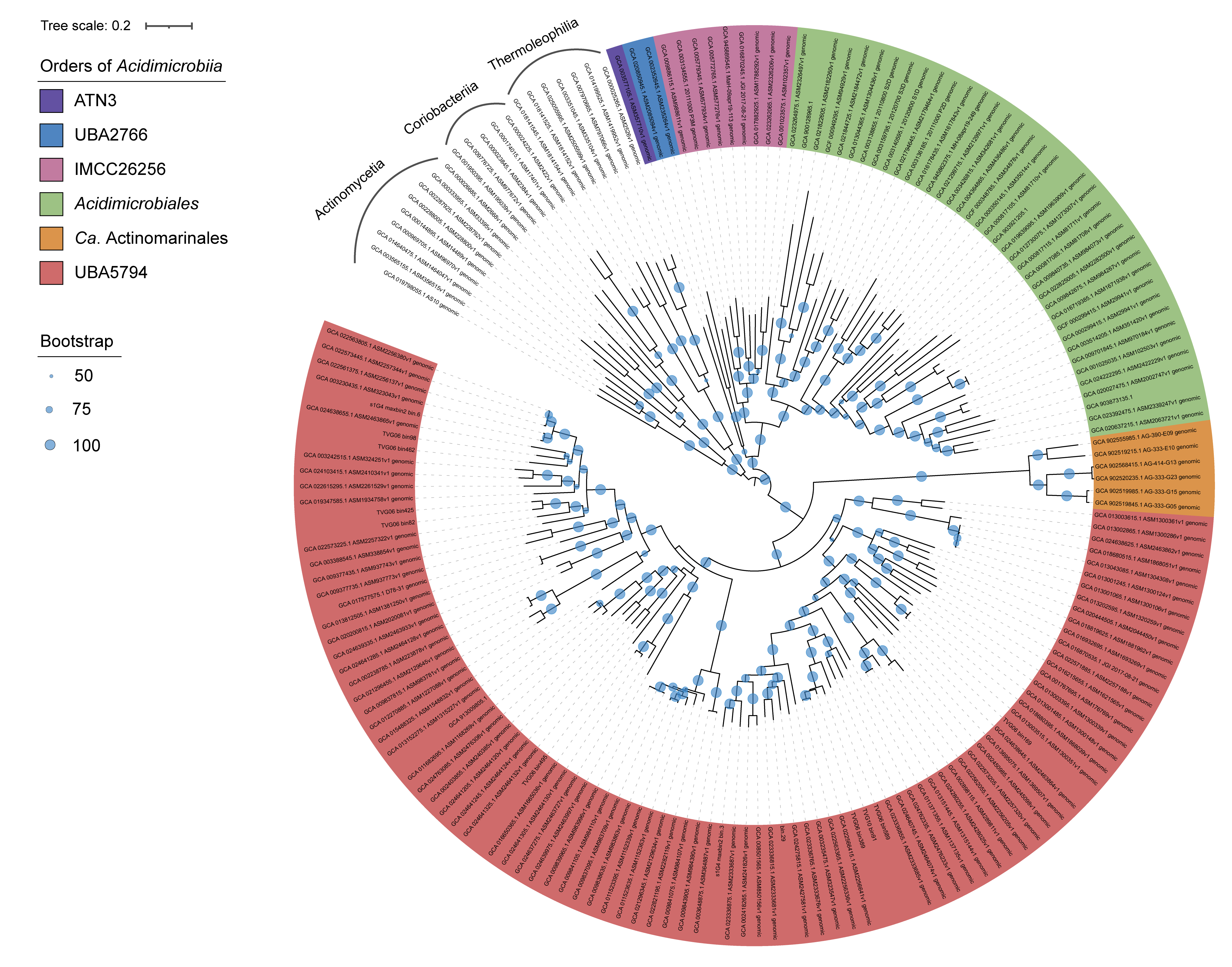

Supplement: Supplementary file 3 — Supplementary Material 3: Figure S3. Genome-wide phylogenetic analysis of class Acidimicrobiia based on the 120 bacterial concatenated marker proteins identified by GTDB-Tk. All the orders of Acidimicrobiia by the GTDB taxonomy are included in this ML phylogenomic tree, with outgroups of nearby actinobacterial classes (Actinomycetes, Coriabacteriia, and Thermoleophilia). Bootstrap values were indicated as dots at branch points. [file 40793_2025_701_MOESM3_ESM.tif]

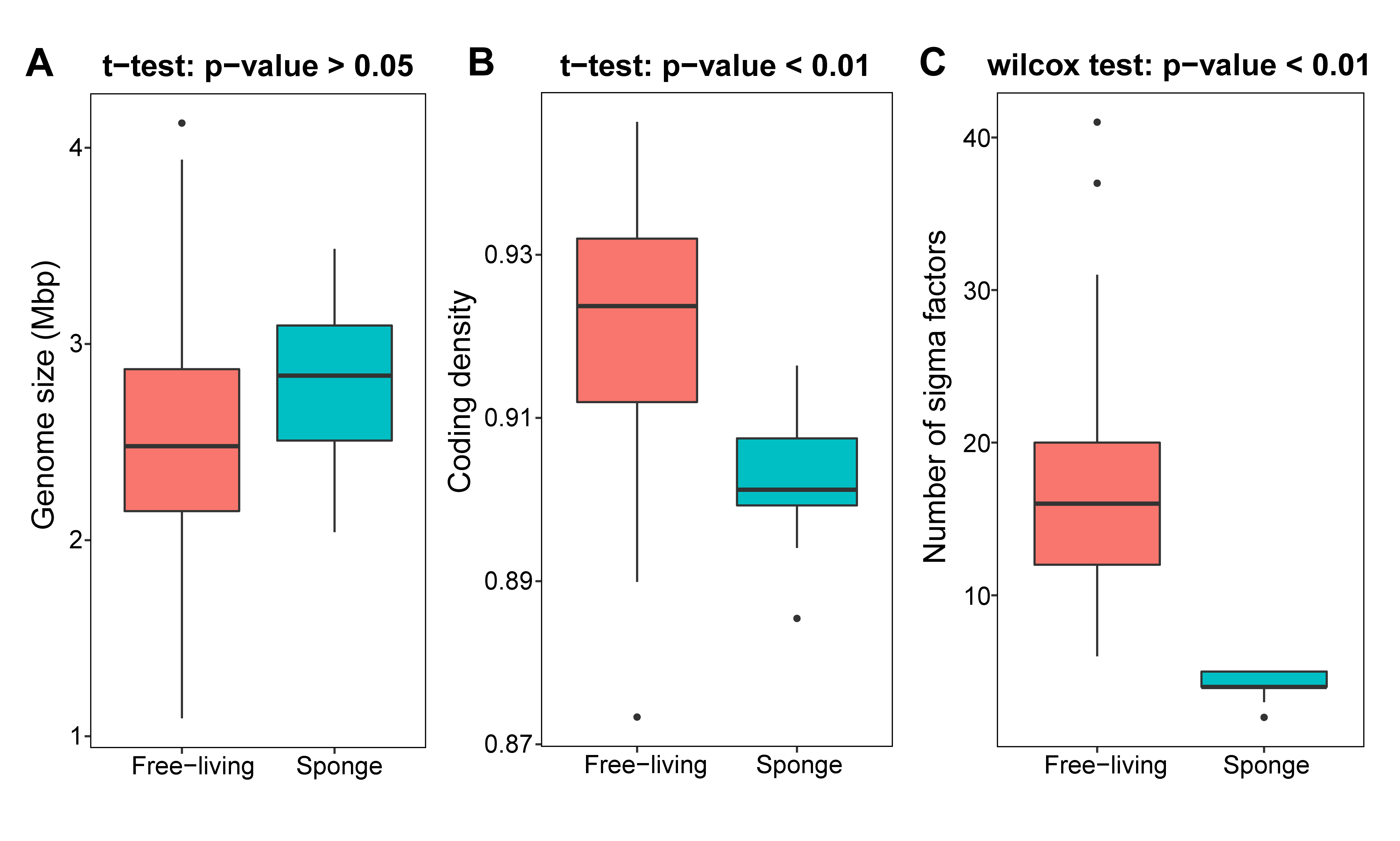

Supplement: Supplementary file 4 — Supplementary Material 4: Figure S4. Statistical comparison of genomic features between free-living and sponge groups. T test was used to determine the difference in genome size (A) and coding density (B) between the two groups. (C) Wilcoxon test was used to determine the difference in the number of sigma factors between the two groups. [file 40793_2025_701_MOESM4_ESM.tif]

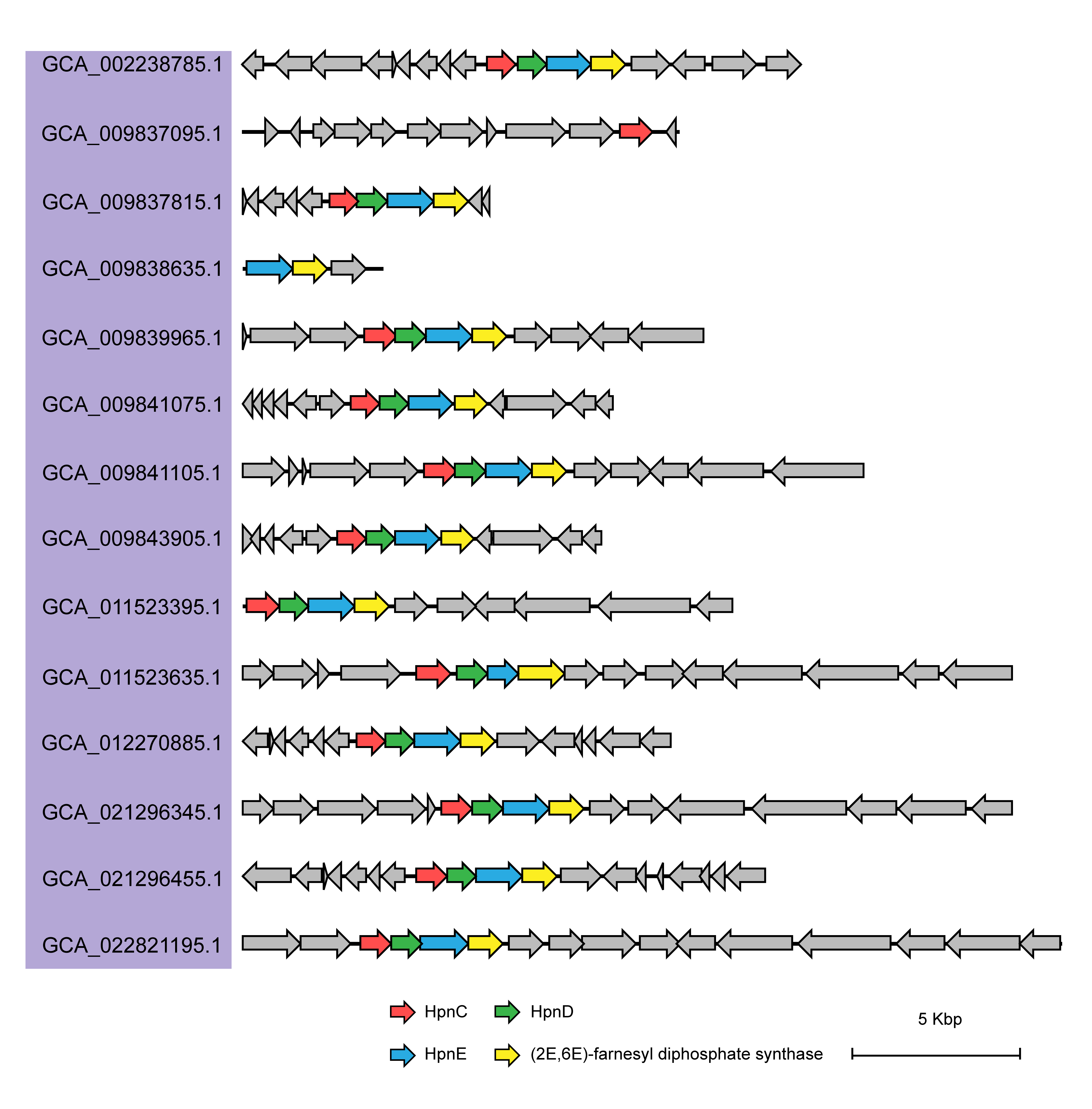

Supplement: Supplementary file 5 — Supplementary Material 5: Figure S5. Squalene biosynthesis gene clusters in UBA5794 genomes from the sponge lineages. [file 40793_2025_701_MOESM5_ESM.tif]

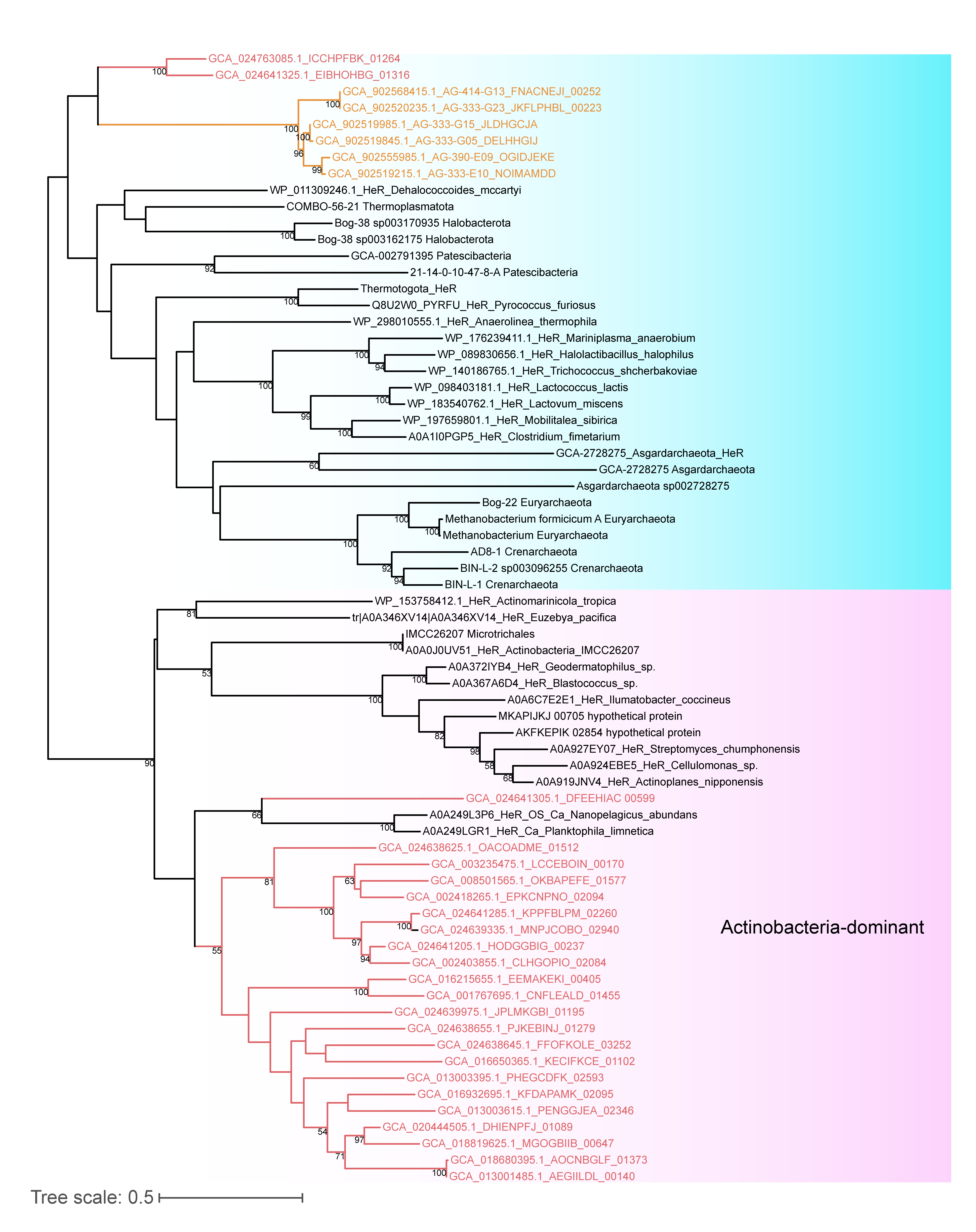

Supplement: Supplementary file 6 — Supplementary Material 6: Figure S6. ML phylogenetic tree of the heliorhodopsin (HeR) protein. HeR sequences from UBA5794 and “Ca. Actinomarinales” genomes were colored in red and orange, respectively. Bootstrap values over 50% were marked at branch nodes. [file 40793_2025_701_MOESM6_ESM.tif]

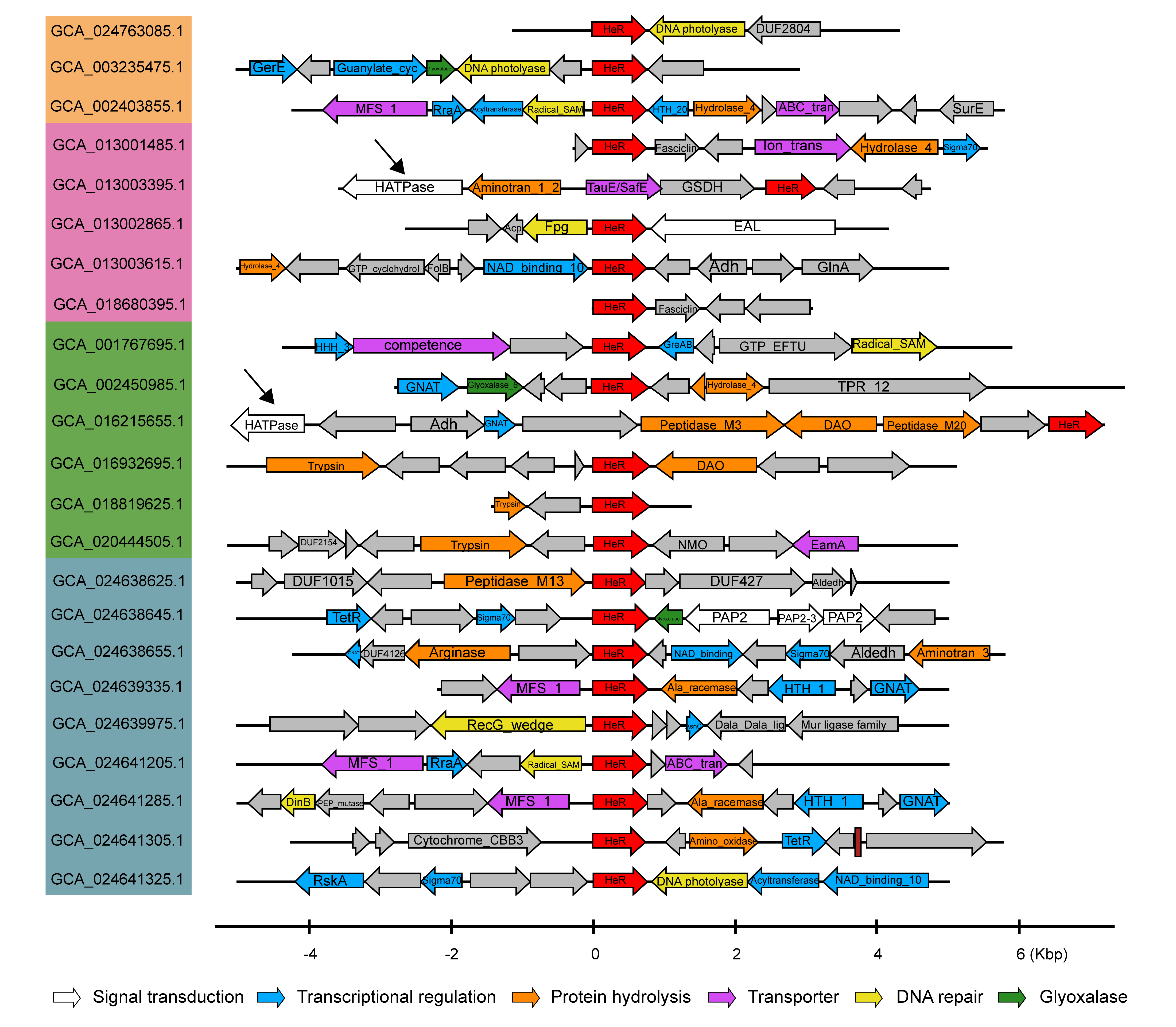

Supplement: Supplementary file 7 — Supplementary Material 7: Figure S7. Organizations of UBA5794 genomic regions including HeR genes and related genes in the neighborhood. Colors in the left column indicate different habitat sources as follows: orange, marine sediments; pink, beach sand; green, inland water systems; blue, brackish sediments. HeR genes were highlighted in red, and the genes coding for protein domains in the vicinity are colored in accordance with functional classification. [file 40793_2025_701_MOESM7_ESM.tif]

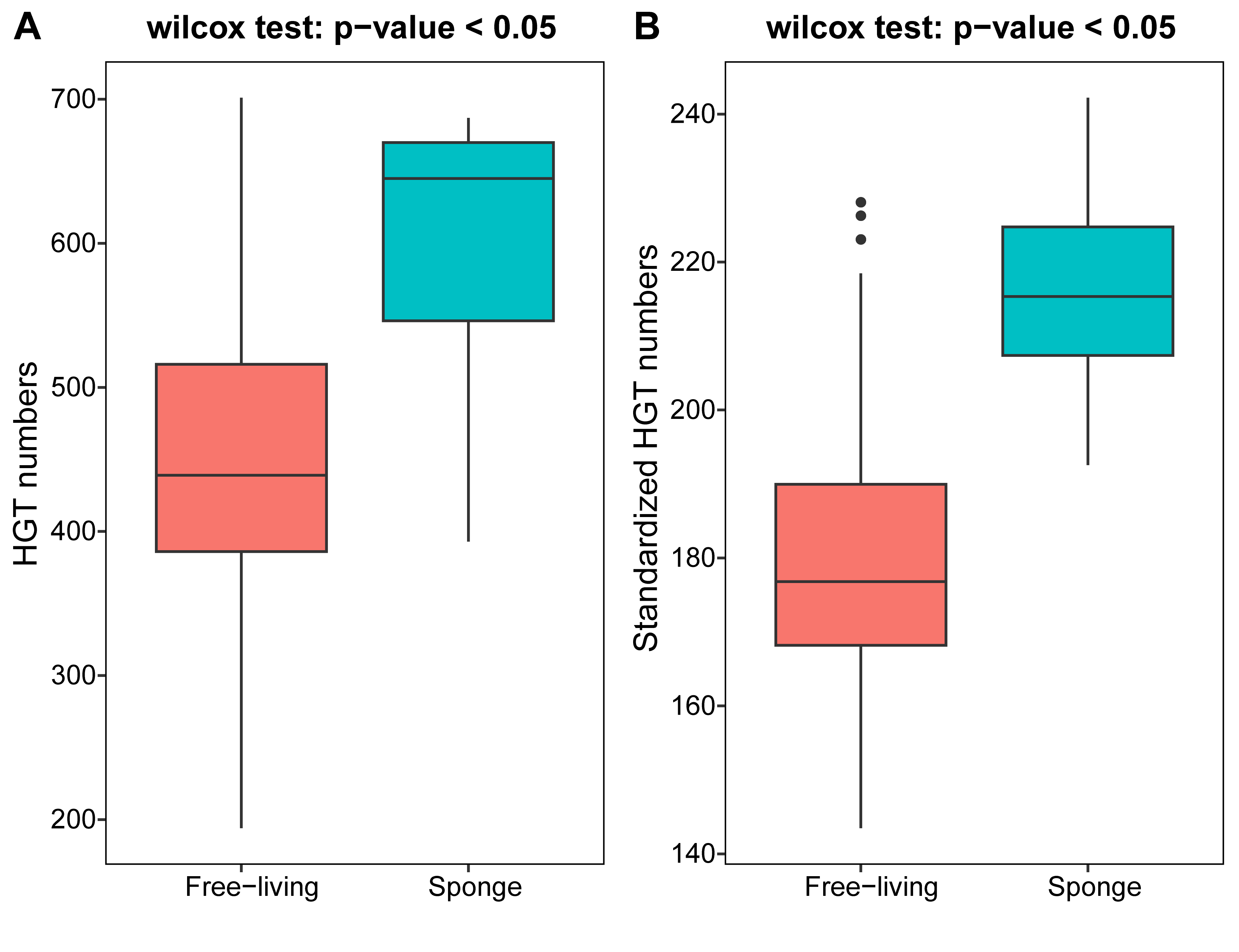

Supplement: Supplementary file 8 — Supplementary Material 8: Figure S8. Statistical comparison of predicted HGT numbers between free-living and sponge derived genomes. Wilcoxon test was used to determine the difference in HGT numbers (A) and standardized HGT numbers (the rate of predicted HGT to genome size) (B) between the two groups. [file 40793_2025_701_MOESM8_ESM.tif]

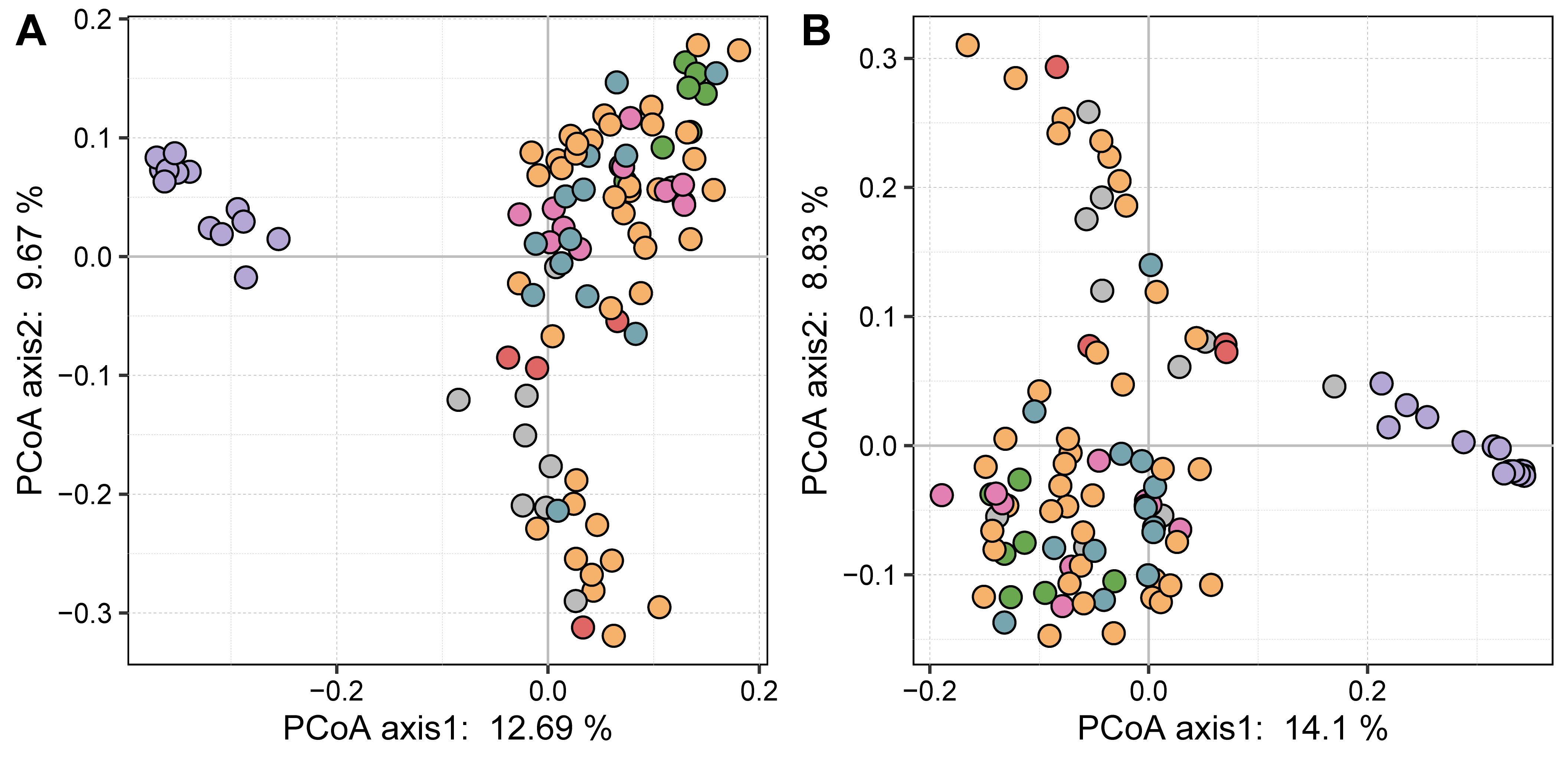

Supplement: Supplementary file 9 — Supplementary Material 9: Figure S9. Ordination of functional annotation of identified HGT genes in 98 non-redundant UBA5794 genomes derived from different habitats. PCoA analysis of (A) KO and (B) PFAM matrices was performed based on Bray-Curtis dissimilarities. [file 40793_2025_701_MOESM9_ESM.tif]

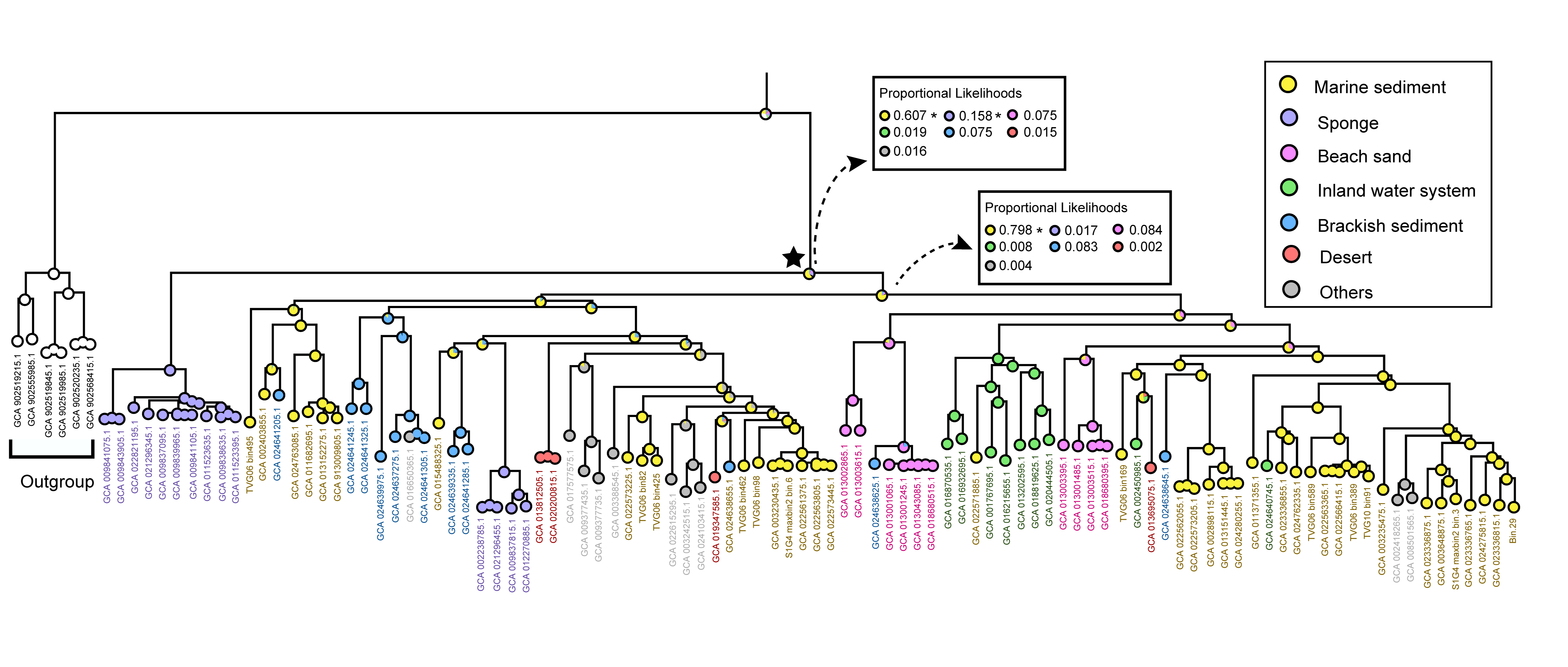

Supplement: Supplementary file 10 — Supplementary Material 10: Figure S10. Ancestral state reconstruction of habitats. Marginal probabilities were calculated based on the habitat states (marine sediment, sponge, beach sand, inland water system, brackish sediment, desert and others) using the Mk1 probability model of likelihood ancestral states. Leaf nodes of the phylogenetic tree based on 291 OGs are colored by the habitat sources, and the colors of internal nodes are displayed in proportion to probability. The MRCA node of UBA5794 is marked with a solid star. Probabilities of the MRCA and the ancestor of cluster 2 are shown in the top right of corresponding nodes. [file 40793_2025_701_MOESM10_ESM.tif]
